# Fluorescence and FTIR Spectra Analysis of *Trans*-A_2_B_2_-Substituted Di- and Tetra-Phenyl Porphyrins

**DOI:** 10.3390/ma3084446

**Published:** 2010-08-23

**Authors:** Pınar Şen, Catherine Hirel, Chantal Andraud, Christophe Aronica, Yann Bretonnière, Abdelsalam Mohammed, Hans Ågren, Boris Minaev, Valentina Minaeva, Gleb Baryshnikov, Hung-Hsun Lee, Julien Duboisset, Mikael Lindgren

**Affiliations:** 1 Gebze Institute of Technology, Department of Chemistry, P.O. Box 141, 41400 Gebze, Kocaeli, Turkey; 2 Université de Lyon, Laboratoire de Chimie de l’ENS Lyon, UMR 5182 CNRS-ENS Lyon, 46 allée d’Italie, 69364 Lyon, France; E-Mails: chantal.andraud@ens-lyon.fr (C.A.); yann.bretonniere@ens-lyon.fr (Y.B.); 3 Theoretical Chemistry, Royal Institute of Technology, SE-10044 Stockholm, Sweden; E-Mails: salam@theochem.kth.se (A.M.); agren@theochem.kth.se (H.A.);; 4 B. Khmelnitski national University, Cherkassy, Ukraine; E-Mail: bfmin@rambler.ru (B.M.); 5 IFM, Linköpings Universitet, SE-58183 Linköping, Sweden; E-Mail: hunle@ifm.liu.se (H.H.L.); 6 Department of Physics, Norwegian University of Science and Technology, NO-7491, Trondheim, Norway; E-Mails: julien.duboisset@ntnu.no (J.D.); mikael.lindgren@ntnu.no (M.L.)

**Keywords:** tetraphenyl porphyrin, asymmetric substitution, electronic structure, TD DFT, FTIR, fluorescence

## Abstract

A series of asymmetrically substituted free-base di- and tetra-phenylporphyrins and the associated Zn-phenylporphyrins were synthesized and studied by X-ray diffraction, NMR, infrared, electronic absorption spectra, as well as fluorescence emission spectroscopy, along with theoretical simulations of the electronic and vibration structures. The synthesis selectively afforded *trans*-A_2_B_2_ porphyrins, without scrambling observed, where the AA and BB were taken as donor- and acceptor-substituted phenyl groups. The combined results point to similar properties to symmetrically substituted porphyrins reported in the literature. The differences in FTIR and fluorescence were analyzed by means of detailed density functional theory (DFT) calculations. The X-ray diffraction analysis for single crystals of zinc-containing porphyrins revealed small deviations from planarity for the porphyrin core in perfect agreement with the DFT optimized structures. All calculated vibrational modes (2162 modes for all six compounds studied) were found and fully characterized and assigned to the observed FTIR spectra. The most intense IR bands are discussed in connection with the generic similarity and differences of calculated normal modes. Absorption spectra of all compounds in the UV and visible regions show the typical *ethio* type feature of *meso*-tetraarylporphyrins with a very intense Soret band and weak Q bands of decreasing intensity. In diphenyl derivatives, the presence of only two phenyl rings causes a pronounced hypsochromic shift of all bands in the absorption spectra. Time-dependent DFT calculations revealed some peculiarities in the electronic excited states structure and connected them with vibronic bands in the absorption and fluorescence spectra from associated vibrational sublevels.

## 1. Introduction

**Figure 1:**
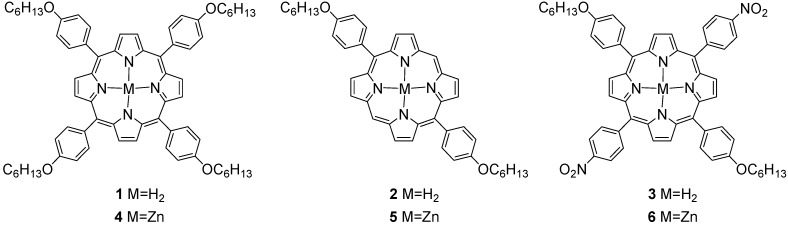
Molecular structures of the di- and tetra-phenyl porphyrins.

Porphyrins are important chromophores that play a crucial role in a number of biological processes such as photosynthesis, dioxygen transport, and activation [1,2]. Moreover, due to their remarkable and quite flexible photo-physical properties, they have been extensively developed for various bio-applications, the most successful of which is perhaps photodynamic cancer therapy [3,4,5]. However, related systems are also being developed for engineering applications for inorganic/organic hybrid materials [6] such as optical power limiting [7,8], photovoltaics [9] and chemical sensors [10,11]. Porphyrin photochemistry provides insight into the dynamics of related biomolecules, such as the photosynthetic reaction centers in purple bacteria and green plants and heme-based metalloproteins such as hemoglobin and myoglobin. Thus, the study of excited states of porphyrins and their vibronic relaxation is important to understand their electronic structure in the context of various applications. Much of this work has recently been focused on free-base and metalloporphyrin assemblies for light-harvesting purposes, porphyrin containing mimics of the photosynthetic reaction center, and electronic devices. Moreover, directed functionalization by attaching pendant groups such as thiols and dendrimers allows the introduction of the porphyrin photochemistry into new material classes such as dendrimers [12,13] and self-assembled monolayers [14,15]. In order to optimize the functionalization of the porphyrine ring, it is important to know the effect upon substitution. We are particularly interested in how the vibration substructure of ABAB substituted diphenyl and tetraphenyl‑porphyrines (DPPs and TPPs), with donor and acceptor moieties, affects the excited states and relaxation mechanisms, such as internal conversion within the singlet manifold, as well as the intersystem crossing of metal substituted variants. A scheme indicating the substitution strategy is presented in Figure 1.

Reliable interpretation and prediction of molecular electronic vibrational spectra requires implementation of quantum chemical methods [16,17,18,19]. The result of a detailed FTIR study is presented and the vibration spectra are analyzed based on the results of advanced quantum chemical calculations. This work is the initiation of systematic research of similar substituted porphyrins with different central ions which will be used as non-linear optical materials and sensors for oxygen detection. With recent progress in developing powerful software for the useful approximate functionals in the density functional theory, the DFT calculations [19,20,21] have become a popular approach for the study of molecular structure, chemical reactivity, phosphorescence, force fields and infrared (IR) absorption spectra [16,17,18]. The hybrid B3LYP functional has been reported to provide excellent thermodynamic parameters and vibrational frequencies of organic compounds if the calculated frequencies are scaled to compensate for the anharmonicity, for basis set and electron correlation treatment deficiencies [16,17,18,19].

## 2. Results and Discussion

### 2.1. Preparation of Asymmetric Di- and Tetra-phenyl Porphyrin Compounds

All porphyrins **1–6** (Figure 1) were obtained by the method developed by Lindsey *et al*. [22] using trifluoroacetic acid and diluted solution of starting materials (10 mM) in dichloromethane. A*_4_* symmetric porphyrin **1** was obtained in one step from 4-(hexyloxy)benzaldehyde and pyrrole, whereas *trans*-A*_2_*B*_2_* porphyrins **2** and **3** were obtained from 4-(hexyloxy)benzaldehyde and known dipyrromethane [23] and 5-(4-nitrophenyl)dipyrromethane [24], respectively. This method selectively afforded *trans*-A_2_B_2_ porphyrins without scrambling, albeit in low overall yield (15 to 20 %). The zinc (II) complexes were readily obtained from the corresponding free base and zinc acetate in refluxing methanol with the eventual addition of few drops of DBU (1,8‑Diazabicyclo[5.4.0]undec 7‑ene) as a base. All compounds were characterized by NMR spectroscopy, mass spectrometry and absorption spectroscopy and their structures unambiguously assigned by ^1^H and ^13^C NMR spectroscopy (see Section 3 for experimental details). The D*_2h_* symmetry of *trans*- A*_2_*B*_2_* molecules gives specific signal patterns with two signals for C_β_-H at 8.91 and 8.87 ppm in compound **3** and 9.36 ppm and 9.09 ppm for compound **2**, with a singlet at 10.27 ppm for the remaining *meso*‑proton for the latter. For compounds **1** and **3**, the upfield shift of the resonance of the C_β_-H protons with respect to porphyrin (9.74 ppm) is similar to what is observed in H_2_TPP (8.70 ppm) [25,26] and is due to shielding effects of the four *meso*-phenyl rings. This effect is less pronounced in compound **2**, as only two meso-phenyl rings participate to the shielding. Central NH protons are also sensitive to the number and the electronic nature of the *meso*-phenyl rings. The NH chemical shifts vary from −3.09 ppm in compound **2** to −2.75 and ‑2.78 ppm in **1** and **3** respectively, intermediate values between −2.07 ppm for H_2_TPP and −3.76 ppm for porphyrin [25]. At room temperature in compound **1** and **2**, one broad signal is observed indicating rapid exchange between the four nitrogen atoms. In compound **3,** the signal is sharper and clearly split in two, which suggests a slower exchange and non equivalent nitrogen atoms because of the strong difference in the phenyl substituents. In the ^13^C NMR spectra, the signals of both C_α_ and C_β_ could not be observed in the A*_4_* compound as well as the C_α_ signals in the two *trans*-A*_2_*B*_2_* compounds. This is a common feature in symmetric tetraphenylporphyrins and was explained by a large broadening of these signals attributable to NH tautomerism [26]. The spectra of the metalated species do not present any unusual features and are very similar to the free bases. The increase of symmetry resulting from the metalation is characterized by the observation of sharp signals for both C_α_ and C_β_ in **4** and two signals for the C_α_ and the C_β_ carbons in the two *trans*-A*_2_*B*_2_* complexes **5** and **6**.

**Table 1:** Crystal data and selected structure refinement parameters for zinc complexes **4**, **5** and **6**.

	4	5	6
Formula	C_68_H_76_N_4_O_4_Zn_1_	C_22_H_22_N_2_OZn_0.5_0.5	C_58_H_52_Cl_6_N_6_O_6_Zn
f.w. (g.mol^-1^)	1078.77	363.12	1207.18
Cryst. Syst.	Orthorhombic	Triclinic	Triclinic
Space group	*Pbcn* (No. 60)	*P-1* (No. 2)	*P-1* (No. 2)
a (Å)	18.923 (5)	9.721 (5)	10.877 (5)
b (Å)	11.138 (5)	10.593 (5)	13.726 (5)
c (Å)	28.542 (5)	10.679 (5)	18.728 (5)
α (^o^)	90	64.838 (5)	88.050 (5)
β (^o^)	90	67.222 (5)	87.781 (5)
γ (^o^)	90	89.723 (5)	84.908 (5)
V (Å^3^)	6016 (3)	900.7 (8)	2782 (2)
Z	4	2	2
T (K)	300	293	120
D_x_ (g.cm^-3^)	1.191	1.339	1.441
μ (mm^-1^)	0.46	0.73	3.73
R(F) ^a^, I>2σ(Fo)	0.064	0.117	0.139
R_w_(F^2^) ^b^, I>2σ(Fo)	0.201	0.247	0.143
S	0.95	1.03	1.05
Rint	0.033	0.044	0.066
θmax	29.2°	29.4	62.8°
Δρ_min_	-0.84 e Å^-1^	-1.32 e Å^-1^	-0.19 e Å^-1^
Δρ_max_	0.85 e Å^-1^	4.70 e Å^-1^	0.19 e Å^-1^

^a^ R(F)=Σ ||Fo|- |Fc||/Σ |Fo|, ^b^ R_w_(F)= Σ[w ((Fo^2^-Fc^2^)^2^/Σ wFo^4^]^1/2^

**Figure 2:**
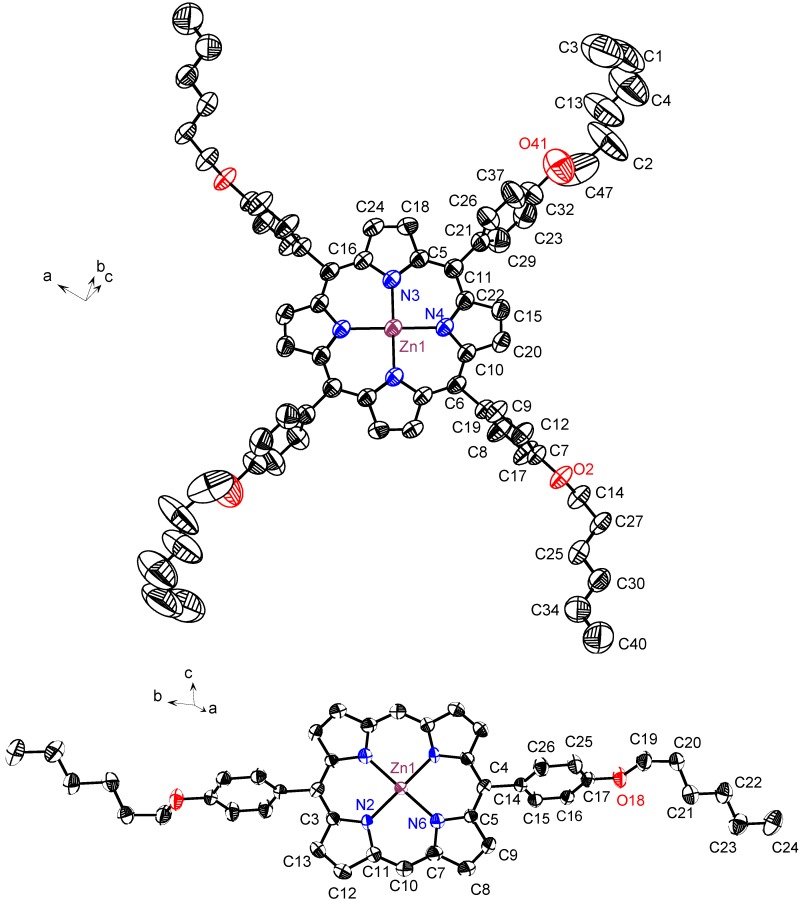
ORTEP view of complex **4** (top) and **5** (lower), with thermal ellipsoids at the 50% probability level. H-atoms have been removed for clarity (note: the atom numbering is different from Figure 3 and refers to the bond distances and angles of Table S2).

Single crystals of zinc complexes suitable for X-ray diffraction (λ_MoKα_ = 0.71069 Å) analysis were obtained by slow evaporation of either a chloroform (**4** and **6**) or toluene solution (**5**). Representative crystal structures **4** and **5** are shown in Figure 2, and all crystallographic data for the compounds **4**–**6** are given in Table 1. Selected interatomic distances are given in Table S1 in the supplementary materials.

**4** crystallizes in the orthorhombic P_bcn_ space group with an asymmetric part that contains two pyrrole rings, as is the case for the crystal structure of the free base [27,28]. Complexes **5** and **6** of the *trans*-A*_2_*B*_2_* porphyrins crystallize in the triclinic P_-1_ space group. For **5**, the asymmetric unit contains a full molecule which is made up of two half molecules together with one solvent molecule (CHCl_3_) for compound **6**. In all three compounds, the porphyrin core composed of 24 atoms is planar with only small deviations from planarity (average deviation are 0.02 Å and 0.05 Å for **4** and **5,** respectively, and 0.05 Å and 0.06 Å for the two units of **6**). Our DFT calculations provide optimized structures with all vibration frequencies real. For Zn-containing porphyrins (4–6) the core is almost planar in agreement with the X-ray data. The larger deviation from planarity is predicted for compound **6**: many carbon atoms have deviations in the range 0.06–0.08 Å, but some C_m_ meso-atoms deviate up to 0.18 Å, which is slightly larger than that provided by the X-ray diffraction analysis for the average out-of-plain deviation.

The zinc atom lies in the plane of the macrocycle and is four-coordinate, on a perfectly square planar geometry in all compounds. Surprisingly, the nitro group does not coordinate to the zinc ion of another molecule as was observed in other zinc complexes of *trans*-A_2_B_2_ porphyrins bearing nitro groups [28]. The average N-Zn-N bond angle equal to 89.5° is found from the X-ray analysis; our DFT calculation provides 88.7° for the block of two pyrrole rings between which the substituent is attached in compound **5** (the next block provides 91.3° as expected). The Zn-N bond length ranged from 2.020 Å to 2.044 Å in our X-ray diffraction analysis to be compared with the 2.037 Å found in the four-coordinate Zn(II) ion in Zn(TPP) [29,30,31,32]. The DFT calculations give the Zn-N bond length ranged from 2.054 Å (compounds 4,5) to 2.056 Å in compound **6**. The phenyl rings attached to the *meso* carbon make dihedral angles of 82.3° and 85.3° for compound **4**, 68.5° for compound **5** and 73.8° and 86.9° respectively for the phenyl ring bearing the nitro group and for the phenyl ring bearing the alkoxy group in compound **6**. DFT calculations reproduce all these trends; the largest deviation from planarity of phenyl rings is predicted for compound **4** (69.5°). Thus, our DFT optimized structures are in a good agreement with the X-ray diffraction data.

### 2.2. FTIR Spectra and Analysis of Vibration Structure

**Figure 3:**
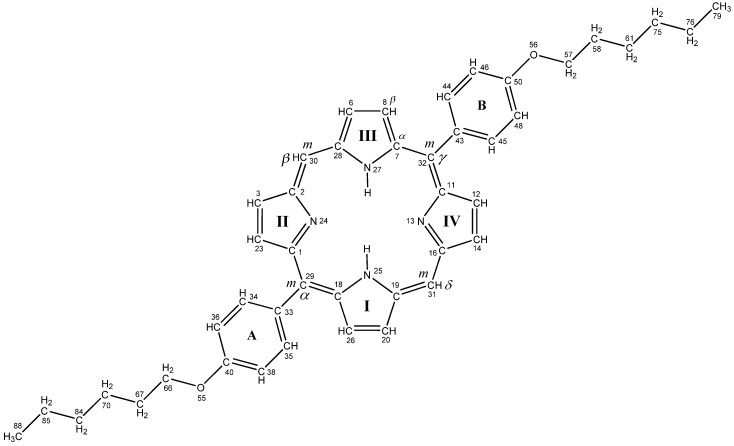
Labeling diagram of 10,20-bis(4-hexoxyphenyl)-porphyrin in TD DFT calculations for FTIR analysis. *m*_α_, *m*_β_, *m*_γ_, *m*_δ_ – are numbers for *meso-*carbon (*m*) atoms, accepted for all studied porphyrin derivatives, C_α_, C_β_ refer to all pyrrole rings.

FTIR spectra, together with luminescence data, gives an accurate description of the geometric and electronic structure of the asymmetrically substituted trans A_2_B_2_-TPPs and DPPs. FTIR spectra were recorded using the standard of the solute molecules dispersed in KBr with details outlined in the experimental section. The aim of this section is to present the DFT study of the newly synthesized non-symmetric derivative of free-base porphin in more detail: 10,20‑*bis*(4 hexoxyphenyl)‑porphyrin (Figure 1) and its Zn complex with a complete interpretation of their vibrational IR spectrum. A comparison with other compounds is also presented. The most asymmetric compound **2** is chosen for numeration of atoms in the porphyrin ring (Figure 3), which is used in the calculations and analysis of fluorescence and FTIR spectra of all compounds.

#### 2.1.1. General appearance of FTIR spectra

**Figure 4:**
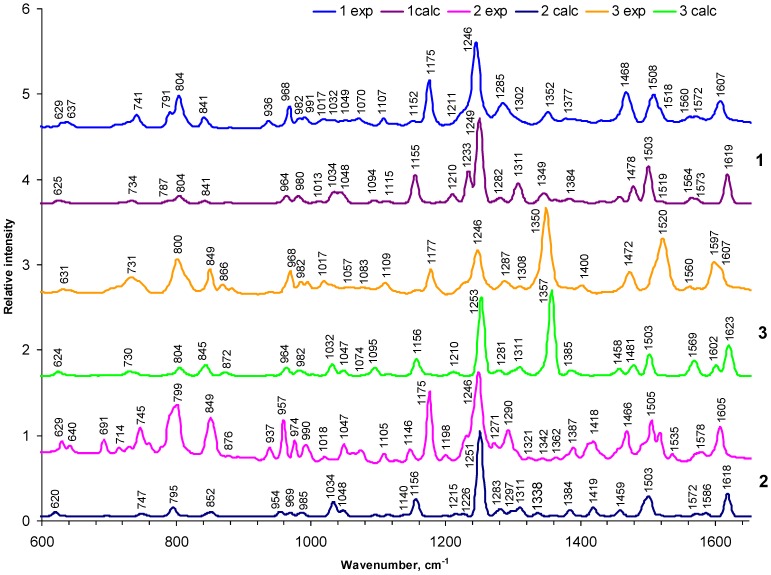
A comparison of the experimental and calculated IR spectra of the investigated tetra- and di-phenyl porphyrins (compounds **1–3**).

**Figure 5:**
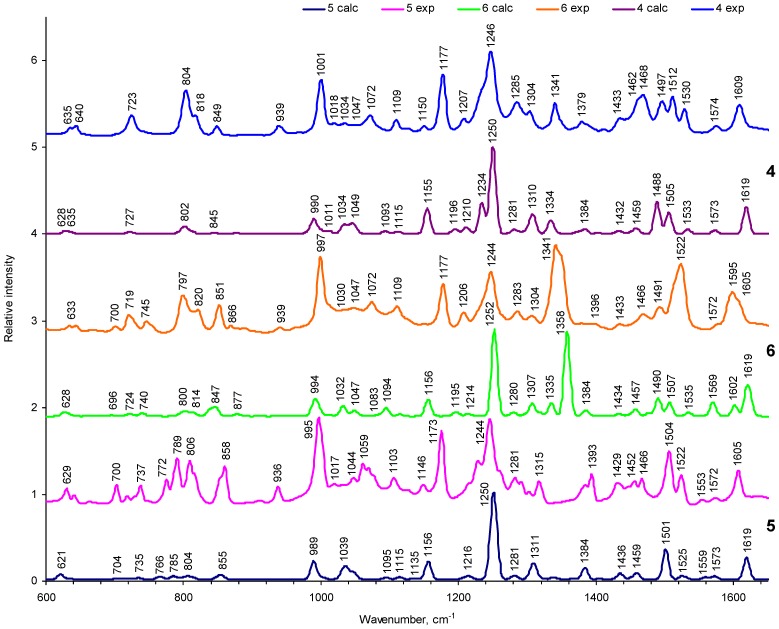
A comparison of the experimental and calculated IR spectra of investigated tetra-and di-phenyl porphyrins complexes with Zink ion (compounds **4–6**).

Results of experimental and theoretical studies of the IR spectra of the 10,20-*bis*(4-hexoxyphenyl)-porphyrin and of its complex with Zn(II) ion (compounds **2** and **5**, respectively) are presented in [33,34]. Figure 4, Figure 5 and Tables S2-S4 in the supplementary material represent some IR spectra calculated by DFT (with scaling factor 0.9745) for all studied compounds in comparison with the experimental FTIR data. We use a consistent numeration of all modes starting from the lowest frequency mode, as it follows from a complete diagonalization of the Hessian calculated with the DFT approach. Though a number of experimental and DFT studies have been published about IR and Raman spectra of asymmetrically substituted di-phenyl and tetraphenyl porphyrins, some key questions still remain unsolved without systematic study [18,35,36,37,38,39,40,41]. The most important question concerns the normal modes which could be strongly affected by asymmetric substitutions.

In all spectra, except the nitro-group-containing compounds **3** and **6**, the IR absorption is dominated by the ether-bridge modes. This is depicted in Figure 4 and Figure 5 where the dominant peak at 1246 cm^–1^ is clearly seen, in agreement with experimental data, which we attribute to the C-O-C vibrations. Even this narrow peak corresponds to a few close-lying modes of a complicated nature, since the C-O-C stretchings additionally include some particular C–N vibrations of the porphyrin ring and small deformations of the methine bridges (Tables S2-S4. SM, modes 271, 269 in compounds **1** and **4**; modes 173, 171 in compound **5**). These are mostly asymmetric vibrations of the C-O-C bridges; in compound **5**, for example, the modes 173 and 171 have a calculated IR absorption intensity equal to 398 and 752 km/mole respectively, which provides a very intense peak (Table S2. SM, see also Figure 5) for these two assymmetrical C-O-C modes. Their symmetric counterparts (modes 170 and 172 in Table S2) have very similar frequencies and are almost forbidden in the IR spectra, but they have a very high activity in Raman scattering. In symmetric tetraphenyl-compounds **1** and **4** there are many vibrations of the C-O-C type: the gap between 269 and 264 intense modes in compound **1** is equal to 16 cm^-1^. That is why the C-O-C band is split at this point and the experimental FTIR spectrum shows a wide shoulder (Figure 4).

The Zn-porphyrins spectra indicate that the Zn(II) ion introduction to free-base porphyrins does not greatly change the C-O-C modes. At the same time, some porphyrin-ring vibrations (for example 188,189 modes in compounds **2** and **5**, Table S2) are interchanged. This example illustrates peculiarities of our study: some simple features of the FTIR spectra can be assigned based on well‑known recommendations from Bellamy and others [43,44]. However, a comprehensive understanding of all vibrational features in the FTIR spectra requires a complete analysis of all normal vibrations on the ground of *ab initio* force field.

In total there are 455 and 452 modes in compounds **1** and **4**; 282 and 278 normal modes in compounds **2** and **5**; and 354 and 351 modes in compounds **3** and **6**, respectively, and the greater part of them are collected and interpreted in Tables S2-S4. The IR spectra of each pair of the relative molecules are quite similar (Figure 4, Figure 5), but some important obvious differences could be very informative, if they are properly assigned. Figure 4 and Figure 5 show the experimental FTIR data and theoretical DFT results for the most important finger print spectral region (600–1650 cm^-1^); high frequency (4000–2900 cm^–1^) of compounds **2**, **5** are analyzed in [33]. In the experimental and theoretical DFT spectra of Figure 4 and Figure 5, the maximum intensity (km/mol in Tables S2-S4, SM) is renormalized to unity.

Valence C–H and N–H vibrations in the high-frequency region for compounds **2** and **5** are discussed elsewhere [33]. In compounds **1–3,** the in-phase stretching vibration of the N–H bonds is predicted at the highest frequency (282 mode in compound **2**, ν_calc._ = 3343 сm^–1^) and is not apparent in the FTIR spectrum because of its negligible intensity (I = 0.01 km/mol). The strong IR band of NH stretching occurs at 3292 сm^–1^. The wide wing in this region of the observed FTIR spectrum [33] is determined by water absorption broadened by intermolecular hydrogen bonding. Similar wide band is seen in compounds **1–3** [33]. Stretching vibrations of the C–H groups are interesting because of their role in non-radiative transitions in porphyrins. In the FTIR spectrum of compound **2,** there are 44 stretching vibration modes of the C–H groups (280–237 modes) in the interval 3117–2843 сm^–1^ [33]. The highest frequencies correspond to symmetric stretching C_β_–H vibrations of the pyrrole rings (3117–3105 сm^–1^). The asymmetric stretching C_β_–H vibrations are detected in the range 3089–3072 сm^–1^. The IR absorption intensity of the C_β_–H vibrational modes in the **II**, **IV** pyrrole rings, which do not contain NH-groups, is higher than in the protonated **I**, **III** rings [33].

The calculated symmetric stretching C–H vibrations in benzene rings of hexoxyphenyl substituents are predicted in the interval 3063–3051 сm^–1^, and their antisymmetric counterparts in the narrow region 3035–3032 cm^–1^. These vibrations in turn occur in the **A** and **B** rings. Intensity of the stretching C‑H vibrations in benzene rings is about two times higher than the intensity of the C_β_–H vibrational modes in the pyrrole rings. In the observed IR spectrum, the C–H bands of benzene rings are seen as a weak absorption in the range 3000–3200 сm^–1^. The more intense absorption in the range 3000–2800 cm^–1^ (in all compounds) is determined by stretching C–H vibrations in methyl- and methylene groups of the hexoxyphenyl substituents. There are many mixings between CH_3_ and CH_2_ alkyl stretchings in all the intense bands of all compounds. After consideration of the C–H valence modes,, we shall shift to the middle IR region 1700–900 cm^–1^ (Figure 4, Figure 5) which corresponds to the “finger-print” modes.

#### 2.2.2. Vibrations of phenyl and pyrrole rings

The phenyl modes are indicated in the FTIR spectrum of all compounds as three bands in the range 1600–1500 cm^–1^ (Tables S2-S4 in supplementary materials, and Figure 4 and Figure 5). An intense peak at 1605 cm^–1^ (Figure 4, compound **2**) is determined by symmetric C=C stretching in both rings being out-of-phase combinations in respect to **A** and **B** moieties (Figure 3, Table S2). A quite similar in-phase combination is less intense. A small wing at 1578 cm^–1^ (Figure 4) is attributed to the asymmetric skeleton vibrations of the methyn bridges and pyrrole rings of the porphin macrocycle in compound **2** (233 mode, Table S2. SM). The left shoulder of this band is assigned to symmetric C=C stretching being in-phase in both rings (modes 232, 231, ν_calc._ = 1572 cm^–1^). Complicated intense band at 1505 cm^–1^ (Figure 4) also belongs to benzene rings, but includes overlapping of the 225–223 modes: the former (*I* = 258.3 km/mol) belongs to the C=C stretching mixed with δ(CH), and the latter mode (223) belongs to the C_m_–C_α_ and C_β_–C_β_ vibrations of the pyrrole rings. All these bands in the range 1600–1500 cm^–1^ are insensitive to introduction of the Zn^2+^-ion (Figure 5), but their intensities are higher in the Zn-porphyrins.

The behavior of the weak complicated band (1572 cm^–1^) upon introduction of the Zn^2+^-ion (Figure 5) is of special interest, since it includes both the porphyrin and benzene ring modes. In compound **5** they are strongly split by 19 cm^–1^ (modes 230 and 232, Table S2); as follows from our calculation they are split by 14 cm^–1^. All these peculiarities are well resolved in the FTIR spectra (Figure 5). A small peak to the right of the 1505 cm^–1^ band, in the FTIR spectrum of compound **2** (Figure 4), includes mixing vibrations of the porphyrin and of the C_m_–C_benz_ chemical bonds (mode 227, Table S2). The weak band at 1535 cm^–1^ in this spectrum (Figure 4), is not only connected with the porphyrin ring, but also includes the CNC angle and the C_m_–C_benz_ chemical bond stretching (mode 228, Table S2). In the Zn-counterpart (compound **5**), this band is shifted by 13 cm^–1^ and overlapped by a more intense band at 1522 cm^–1^ (mode 227). Interpretation of all other porphyrin ring vibrations coincides with previous works [13,20,21]. Valence vibrations of the C–C bond between benzene ring and porphyrin macrocycle, *ν*(С*_benz_*–C*_m_*), occur in the broad frequency range. For example, in compounds **2** and **5,** we assign them to *ν_calc._*: 1545–1430, 1280–1250, 870 cm^–1^. A very weak band at 555 cm^–1^ (mode 67 in compound **2**; Table S2) is assigned to the out-of-plane deformation vibrations С*_benz_*–C*_m_*, which is mixed with the CH deformation vibrations of benzene rings. This band is shifted by 2 сm^–1^ in the IR spectrum of the Zn-porphyrin.

Mode 228 in compound **2** (calc.: 1545 сm^–1^, exp.: 1535 сm^–1^, *I* = 17.3 km/mol), in which the skeleton *ν*(С*_benz_*–C*_m_*) out-of-phase vibrations are mixed with the valence *ν*(C*_m_*–C_α_), *ν*(C_β_–C_β_) and *ν_s_*(С_α_–N) macrocycle stretching, is down shifted by 13 сm^–1^ under the Zn-complex formation in compound **5**. Absorption bands which are responsible for skeleton vibrations of alkyl fragments in all compounds have low intensity; they occur in the ranges 1122–885 cm^–1^ and below 460 cm^–1^. The first range is determined by C–C skeleton vibrations, the second – by δ(CСС) deformation vibrations. In the Zn complexes they do not show frequency shifts (Tables S2-S4): See, for example, modes 152, 151, 146, 145; Table S2.

#### 2.2.3. Deformation vibrations

The in-plane NH deformation modes are important because of their connection with isomerization. Weak observed bands at 1418, 1198 and 990 сm^–1^ (modes 205, 163 and 129, respectively; Table S2), belong to the deformation vibrations of NH bonds in the IR spectrum of compound **2.** An absence of such bands in the IR spectrum of the Zn-analogous supports this assignment. Besides that, the in-plane NH-deformation vibrations contribution is present in the calculated modes 1586 (233 mode), 1494 (223 mode), 1357 (193 mode), 1204 (164 mode), 969 сm^–1^ 126 mode), determined by vibrations of pyrrole rings. It is also present in the benzene ring vibration at 1297 сm^–1^ (mode 185). These modes are the most sensitive to the metal ion introduction in the coordination center. One should stress that the IR band of compound **2** at 974 сm^–1^ (mode 126), which has also NH bend and pyrrole breathing contributions; is up-shifted by 21 cm^–1^ (995 сm^–1^) due to the disappearance of NH-bonds under the Zn complex formation. Further, its IR intensity increases from 52 to 188 km/mol (including overlap, Table S2). This effect is well predicted by the DFT calculations and is clearly seen in the experimental IR spectra (Figure 4, Figure 5).

The out-of-plane γ(NH) vibrations in free-base porphyrins occur in the experimental IR spectrum as two bands at about 740 and 800 сm^–1^ (modes 138 and 158 in compound **1**, modes 88 and 98 in compound **2**, modes 116 and 130 in compound **3**). The former one is not observed for the Zn complex, but the latter band, which has large contribution from the out-of-plane CH deformation in the pyrrole rings (abr. γ(C_β_H)) and from methyne bridges (abr. γ(C_m_H)), still exists, but its IR intensity diminishes. This intensity loss is much more prominent in compounds **2** and **5** (from 107.3 till 15.2 km/mol with account of overlap, Table S2) than in compounds **3** and **6** (from 98 to 65 km/mol, Table S3), in agreement with observation. The largest changes in IR spectra are observed in the range 745–810, 970–996 cm^–1^, due to disappearance of NH-vibrations for the Zn complexes. Besides this, the band at 957 (**2**) and 964 cm^–1^ (**1**, **3**) disappears, which belongs to deformation in pyrrole rings being mixed with the out-of-plane CH-vibrations in benzene rings (Figures 4, Figures 5). Thus the complicated nature of mixed vibrational modes is supported by observed IR spectra.

The in-plane CH deformations in pyrrole rings (abr. δ(C_β_H)) are often mixed with other vibrations, or their IR bands are overlapped by more intense absorption [18]. The complex band at 1047 cm^–1^ in the experimental IR spectrum of compound **2** is formed by the overlap of eight vibrational modes; four of them (141–144) belong to δ(C_β_H)-vibrations and four others (146, 145 and 140, 139) belong to C–O vibrations of the ether bridges (abr. С_hex_—О). Calculation shows that the largest frequency shift under the Zn complex formation occurs for mode 139; moreover its IR intensity strongly increases which leads to the change of the band profile in the IR spectrum of compound **5,** in respect to the spectrum of **2** and to the up-shift of the band maximum by 12 cm^–1^. The observed bands of intermediate intensity at 849, 799 cm^–1^ and less intense band at 691 cm^–1^ in the IR spectrum of compound **2** are assigned to out‑of‑plane deformation vibrations of the С_β_Н-bonds of pyrrole rings (abr. γ(C_β_H)). The latter band has a contribution of the out-of-plane deformations of carbon and nitrogen atoms of pyrrole rings. The band at 799 сm^–1^ is the most intense, but it loses intensity under the complex formation with Zn ion since γ(NH) vibrations disappear (Figure 4, Figure 5). This effect is less pronounced in more symmetric compounds (Tables S2-S4). The CH-deformation vibrations of methyne bridges in the *meso*‑disubstituted porphins **2**, **5** are mixed with the C_β_H-vibrations and have no particular observable bands in the IR spectra.

CH deformation modes in aromatic rings are well known, but in the studied compounds they have specific peculiarities. The in-plane deformational vibrations of CH-bonds in phenyl rings are calculated to be in the 1300–1100 сm^–1^ range. In the observed IR spectrum they are identified as a strong band at 1175 cm^–1^ and a weak band at 1105 сm^–1^. These bands are quite stable in all compounds. One has to note that our DFT calculations systematically underestimate the δ(CH)‑frequencies in all compounds by 10–20 сm^–1^. The out-of-plane deformational vibrations of CH‑bonds in phenyl rings occur in the range 940–535 сm^–1^. In the IR spectrum of compound **2,** we have found a similar assignment for the weak IR bands at 937, 714, 555 and 534 сm^–1^. Besides the first band, this absorption also includes other types of vibrations; all these bands are strongly shifted in other compounds, but are more stable under the Zn complex formation (Figure 4, Figure 5; Tables S2-S4). Deformation vibrations of alkyl groups usually do not interact with other modes and are not sensitive to the ion introduction. But the wagging vibrations of the methylen groups (modes 199, 180 in compound **2**) are mixed with porphyrin macrocyle (199) and benzene ring vibrations (180). The corresponding absorption bands in the observed IR spectra are up-shifted by 6 and 10 cm^–1^, respectively, upon the complex formation with Zn-ion, though DFT calculations do not reproduce this trend.

#### 2.2.4. The influence of the nitro-group

Compounds **3** and **6** have specific changes in the IR spectra: the occurrence of an intense new band at 1350 cm^–1^ and evident quenching of the O–C–O band (1246 cm^–1^) activity (Figure 4, Figure 5). The former band is attributed to the out-of-phase vibrations of the C–NO_2_ groups (*I =* 980 km/mole) in compound **3** (mode 248). The in-phase counterpart (mode 249) is up-shifted only by 1 cm^–1^ and is less intense (*I =* 259 km/mole; it is strongly mixed with pulsation of the porphyrin ring). Both bands are more equalized in intensity upon Zn-ion introduction in compound **6**. In this complex, two additional modes at 1335 cm^-1^ occur (Table S4), which leads to a more wide absorption with a maximum shift down of 9 cm^-1^ (Figure 5). The O–C–O band, which is dominant in all other compounds (**1**, **2**, **4**, **5**), is now split and is therefore less intense. Since compound **3** includes two modes (*I =* 533 km/mole for mode 222 and *I =* 387 km/mole for mode 221); the intensity of the bands is less (Figure 4, Figure 5, Table S4).

#### 2.2.5. Specific porphyrin ring modes in comparison with other studies

Out-of-plane (oop) vibrations of the macrocycle are assigned to the 723 cm^-1^ and 700 cm^-1^ peaks in compounds **4** and **5**, respectively. This vibration has been assigned to γ_7_ = 706 cm^-1^ according to classification for Zn-tetraphenyl-porphyrin (Zn-TPP) [34]. In the Zn-diphenyl-porphyrin (Zn-DPP) this mode is shifted down to 693 cm^-1^ [34]. This trend is in qualitative agreement with our out di- and tetra-phenyl derivatives. The other and more intense oop deformations of the macrocycle are mixed with the oop γ(C-H) vibrations of phenyl rings; this is γ_5_ = 797 cm^-1^ (783 cm^-1^) according to classification for Zn‑TPP (and for Zn-DPP, respectively) [34]. In our substituted analogous, we found these modes at 804 and 789 cm^-1^ for compounds **4** and **5**, respectively. There are additional splittings of the similar oop deformation vibrations in compound **5** for the C_β_-H and C_m_-H groups (Figure 5). In the region of 1000–1070 cm^-1^, a number of intermediate intensity IR bands are observed for the Zn-complexes (compounds **4–6**) which are shifted to the 960–1030 cm^-1^ range in their free-base analogs (**1–3**). The most intense band here belongs to out-of-phase breathing of pyrrole rings. In all tetraphenyl derivatives (**1**,**4**,**3**,**6**), the out-of-plane porphyrin macrocycle vibrations are mixed with phenyl rings deformation and produce less intense IR absorption than compounds **2** and **5**. The modes which are not active in the IR spectra (a_g_ and b_1g_ modes in tetraphenyl-free-base-porphyrin: H_2_-TPP) are the most important for vibronic activity of absorption and emission in the visible region [42]. That is why we collected (Tables S2-S4) some characteristic vibrations which have very low IR intensity in non-symmetric compounds. The peak at 963 cm^-1,^ in the Raman spectrum of H_2_-TPP, is a pyrrole in-phase breathing, which is well known as ν_6_ vibration according to classification of Oakes and Bell [45]. In free-base porphyrin this is a totally symmetric vibration at 988 cm^-1^ in the IR spectrum [46]. In compound **2** we have assigned it to the 132 mode at 989 cm^-1^ with intensity 3.2 km/mol. This mode gives the onset of the 0–1 band in electronic transition of all compounds. The most intense lines in the 0–1 band are discussed in the next section.

### 2.3. Optical Absorption, Excited States and Fluorescence

**Figure 6:**
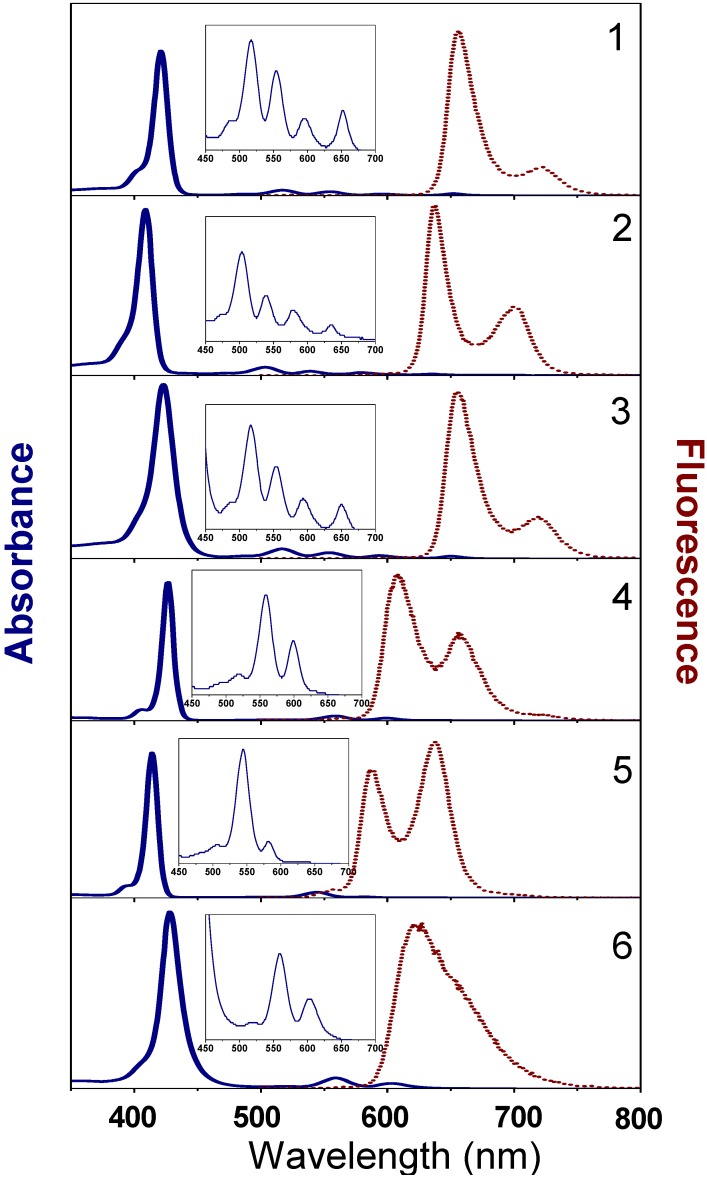
Absorption and emission spectra of the compounds in THF solution. The absorption and fluorescence maxima are collected in Table 2.

**Figure 7:**
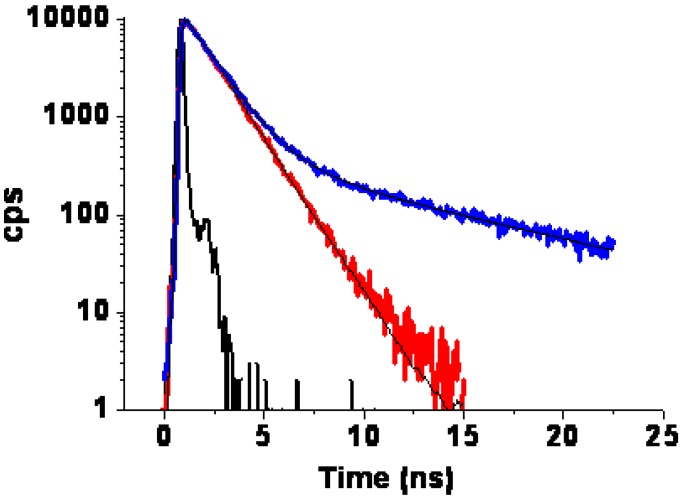
Fluorescence decay of **4** in THF solution for two emission wavelengths: Red 607 nm; Blue 657 nm. Excitation at 405 nm.

Representative absorption and emission spectra, from a detailed luminescence characterization of compounds **1–6** in aerated and degassed THF solutions, are shown in Figure 6, Figure 7 and the key parameters summarized in Table 2. These include the absorption and emission wavelengths, fluorescence quantum yields, and excited state lifetimes. Theoretical results of the excitations and excited states are collected in Table 3.

#### 2.3.1. Theoretical and experimental absorption spectra

The absorption spectra of all free bases (Figure 6, Table 2) show the typical *ethio* type feature of *meso*-tetraarylporphyrins with a very intense Soret band in the wavelength blue to ultraviolet wavelength (350–430 nm) and four weaker Q bands in the green to red region (520–650 nm). The presence of four electron enriched or electron poor phenyl rings does not influence the position of the bands as neither the B band nor the Q bands in compounds **1** and **3** are significantly shifted with respect to H_2_TPP. The presence of only two phenyl rings on the other hand causes a pronounced hypsochromic shift (10 to 15 nm) of all bands. Ongoing from porphyrins to metalloporphyrins, the spectra are generally simplified with only two Q bands due to an increase in the symmetry of the system, as generally observed in other porphyrin systems. This is also the case for the *trans*-A*_2_*B*_2_* compounds studied here and we can conclude that the substitution scheme has a small influence on the central porphyrin unit.

**Table 2:** Photophysical parameters of asymmetrical TPPs in THF.

Sample	Absorbance (nm)	Emission wavelength (nm) [decay time (ns)]	Quantum Efficiency ^a^
**1**	S: 421; Q: 517; 554; 596; 652	656 [4.5]; 721 [4.5]	0.13
**4**	S: 427; Q: 558; 595	607 [1.3]; 657 [1.3 and 8.7]	0.05
**2**	S: 409; Q: 503; 538; 578; 634	637 [5.2]; 700 [5.2]	0.06
**5**	S: 414; Q: 545; 580(w)	587 [2.4]; 638 [2.4]	0.03
**3**	S: 423; Q: 517; 554; 593; 650	655 [5.0]; 720 [5.1]	0.13
**6**	S: 428; Q: 559; 602	622 [1.40]	0.07

^a^ λ_exc_ = 420 nm, except **2** and **5** (λ_exc_ = 410 nm); reference Coumarin 153 in methanol (Φ = 0.45).

These bands are usually explained in terms of the four-orbital scheme of Gouterman [47]. Since the beginning of the DFT era, this old semiempirical theory has been confirmed by numerous TD DFT calculations, see for example [16,48]. In the case of the most simple compound **2,** the computed and measured absorption spectrum (Table 2 and Table 3) indicates a very good agreement with the experimental high-resolution free-base porphyrin counterparts [38,39] over the entire energy range of the S_0_-S_1_ transition (500–640 nm). This corresponds to the so-called Qx and Qy band systems (transitions to the ^1^B_2u_ and ^1^B_1u_ states) each consisting of the 0–0 and 0–1 bands in vapor phase low-resolution absorption spectra [47]. In Zn-complexes there are degenerate ^1^E_u_ excited states (instead of ^1^B_1u_ and ^1^B_2u_ counterparts in free-base porphins) and only one Q band occurs with the 0–0 and 0–1 sub-bands (compare e.g., absorption for **2** and **5** in Figure 6). A similar agreement and corresponding shifts in the absorption and emission spectra of the respective counterparts were obtained for all of the compounds studied here (Figures 6).

**Scheme 1:**
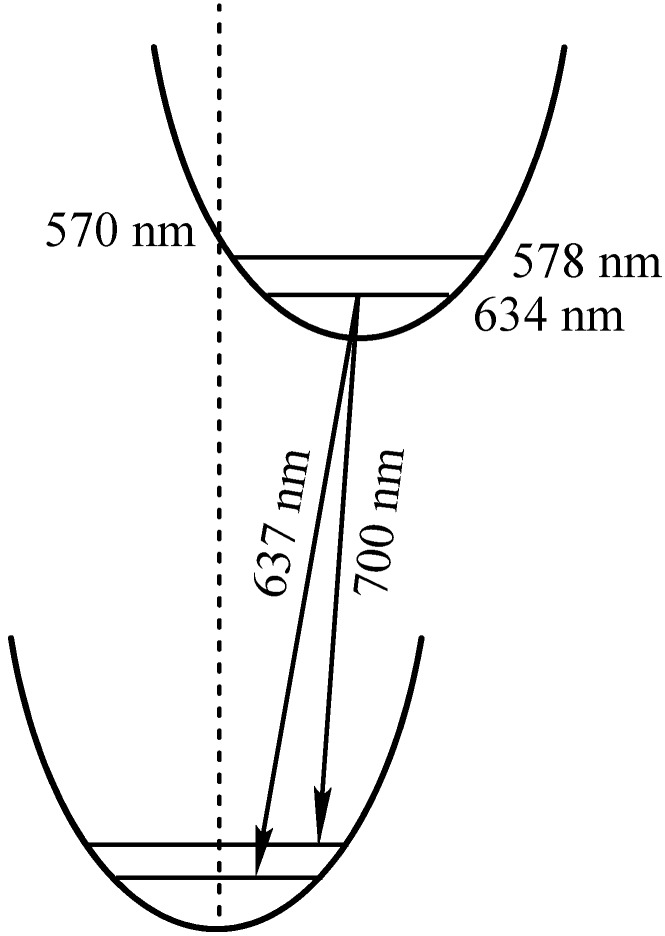
A schematic detailing the ground and first excited singlet state potential curves of compound **2**.

There is no full agreement between experiment (Table 3) and theory (Table 4) for obvious reasons. Our TD-DFT calculations of the vertical transitions from the optimized ground state geometry provide a blue shift (Table 4) in comparison with the observed absorption bands (Figure 6). This is quite natural, taking into account the qualitative view presented in Scheme 1. The first very weak absorption band of compound **2** at 634 nm corresponds to the 0–0 transition. In the free-base porphyrin, this corresponds to the ^1^A_g_-^1^B_2u_ (0–0) adiabatic excitation (Q_x1_ band). Because of the displacement in the excited state equilibrium structure, the calculated vertical transition from the optimized ground state geometry (570 nm, Table 3, Scheme 1) is closer to the observed 0–1 adiabatic excitation (578 nm, or Q_x2_ band), but is still slightly blue shifted (see Scheme 1.). 

The Q_x_ band in free-base porphyrin has been calculated recently taking vibronic perturbations into account [42,49], in good agreement with the observed fine structure in the experimental high‑resolution spectra [38]. As seen in previous studies [42,49] of free-base porphin (FBP) molecules, the vibronically induced transitions by the Herzberg–Teller mechanism (HT) are found to be much more important than the lines determined by Franck–Condon (FC)-factors and the *b*_1*g*_ vibrations appear to be more intense than the totally symmetric modes [42]. The vibronic 0–1 line at 1600 cm^-1^ (this is the *ν*_19_ mode in ref. [40] notations, which corresponds to C_α_‑C_m_ asymmetric stretching vibrations of the b_1g_ symmetry) is active in the electronic Q_x_ transition [42], though this mode is predicted to be very weak in Raman scattering of FBP and is forbidden in the IR spectra. From our DFT calculation, this mode includes asymmetric (out-of-phase) stretchings of C_α_-C_m_ bonds attached to the protonated pyrrole rings in compounds **1–3** with weaker involvement of the other C_α_-C_m_ bonds in agreement with the empirical force field calculations [38,40]. In accordance with the previous vibronic treatment [42], mode *ν*_19_ produces one of the most intense 0–1 lines which form the Q_x_ band of FBP. Together with the *ν*_10_ = 1610 cm^-1^ line of the totally symmetric type, they provide the shorter-wavelength intense wing of the 0–1 vibronic band. In our molecules, these two bands are shifted to lower frequencies (about 1540–1520 cm^-1^), which are seen as a gap (1529 cm^-1^) between 634 and 578 nm in the absorbance spectrum of compound **2**. The large gap of 1565 cm^-1^ observed for compond **3** between the 650 and 590 nm bands, is in agreement with our DFT prediction for the *ν*_10_ and *ν*_19_ modes.

The fluorescence spectra of all compounds consist of two peaks produced by the shorter wavelength 0–0 band and the weaker 0–1 band of the ^1^B_2u_ → ^1^A_g_ transition (in notation of D_2h_ symmetry); in compound **6** the second peak is overlapped by the more intense 0–0 band. In compound **5** the 0–1 band is more intense for unknown reasons. Probably this is connected with deviation from the Jahn-Teller effect in the pseudo ^1^E_u_ state of non-symmetrically trans-substituted porphyrin. The compounds show similar fluorescence decay-times as symmetrical tetraphenyl porphyrins in with dendritic scaffolds [12,13], that is, longer decays in the range 4–5 ns for the proto-forms, and shorter decays in the range 1–2 ns for the same compounds bearing the metal ion (Zn). Representative fluorescence time decays are shown for compound **4** in Figure 7. Except for the long wavelength emission for compound **4** (at 657 nm), all fluorescence measured decays were single exponentials. Data, in terms of decay times for all compounds, are collected in Table 2.

The vibronic 0–1 band in emission is determined by a number of close lying modes in the range 1250–1413 cm^-1^. There is no complete mirror symmetry in absorption and emission spectrum of free-base porphyrin [39,42,49]. This becomes clear from the assignments of the main contributing modes. The two strong 0–1 components in the absorption peak (a_g_ mode 1604 cm^-1^ and b_1g_ mode 1357 cm^-1^ in notations of [42]) have much weaker counterparts in emission due to the interplay of Duschinsky and Herzberg-teller effects [49]. Both previous modes (*ν*_10_ and *ν*_19_) also produced quite intense close-lying features in fluorescence spectra, in agreement with the Q_x_ emission band analysis [39,42]. They are slightly shifted to lower frequency in the absorption spectra, as in this spectra, the vibrational modes of the upper state are slightly lower [39]. This is in good agreement with our FTIR and UV-visible spectra and with DFT calculations for the present **1–6** compounds. One has to note that in the same IR region, there is very intense IR absorption determined by phenyl ring vibrations (*ν*_235_ = 1605 cm^-1^) in the case of compound **2** (Table S2). Being active in the IR spectra, the phenyl modes do not contribute to vibronic bands, neither in absorption, nor in emission, since electronic excitation is localized in the porphyrin ring. Epecially, *ν*_10_ and *ν*_19_ are separated by only 10 cm^-1,^ in free base porphyrin (FBP), whereas, in metalloporphyrins, this separation increases to about 50 cm^-1^ [1,35,36,37,38,39,40]. In compound **2** and **5** the calculated IR bands *ν*_233_ and *ν*_230_ are separated by 27 cm^-1^ in a semiquantitative agreement with analysis of the simpler porphyrins. The reason for such difference in positions of the most active fluorescent bands in FBP and ZnP, for example, is that the interaction between adjacent C_α_-C_m_ and *C*_α_–N bands has a much larger influence on *ν*_19_ in the ZnP molecule than on the *ν*_10_ frequency [40]. This can be used as an explanation for the large difference in the 0–1 band behavior in fluorescence of free-base tetraphenyl porphin (H_2_TPP) and ZnTPP in dendrimers [12,13] and in all compounds analogous to the ones presented as examples in this study (compounds **1–6**).

**Table 3:** Wavelengths (*λ*, nm) of transitions, their energies *E* (eV), assignment and oscillator strengths (*f*) in electronic absorption spectra of compounds **1–6** calculated with B3LYP level of theory.

Compound	№	λ	*E*	F	Assignment
**2**	1	569.9	2.18	0.0206	HOMO→LUMO+1(+45%) HOMO-1→LUMO(+30%)
					HOMO→LUMO(19%) HOMO-1→LUMO+1(+10%)
	2	535.5	2.32	0.0525	HOMO→L+0(+44%) HOMO-1→LUMO+1(27%)
					HOMO→LUMO+1(+16%) HOMO-1→LUMO(+12%)
	3	391.6	3.17	1.1557	HOMO-1→LUMO+1(+24%) HOMO-3→LUMO(18%)
					HOMO-1→LUMO(+9%) HOMO→LUMO(+9%)
					HOMO-5→LUMO(+8%) HOMO-5→LUMO+1(7%)
	4	384.0	3.23	0.5538	HOMO-1→LUMO(+21%) HOMO-3→LUMO(+12%)
					HOMO→LUMO+1(11%) HOMO-3→LUMO+1(+11%)
					HOMO-2→LUMO(+9%) HOMO-5→LUMO+1(6%)
					HOMO-1→LUMO+1(6%)
	5	381.3	3.25	0.0513	HOMO-2→LUMO(+85%)
**5**	1	529.5	2.34	0.0001	HOMO→LUMO(+56%) HOMO-1→LUMO+1(47%)
	2	528.4	2.35	0.0164	HOMO→LUMO+1(+57%) HOMO-1→LUMO(+45%)
	3	386.5	3.21	1.2378	HOMO-3→LUMO+1(+38%) HOMO-1→LUMO(+25%)
					HOMO→LUMO+1(18%)
	4	379.7	3.27	0.3924	HOMO-3→LUMO(31%) HOMO-1→LUMO+1(+24%)
					HOMO→LUMO(+19%) HOMO-2→LUMO+1(5%)
	5	376.1	3.30	0.0201	HOMO-2→LUMO+1(+92%)
	6	371.9	3.33	0.0003	HOMO-2→LUMO(+98%)
	7	358.8	3.46	0.0000	HOMO-4→LUMO(+95%)
	8	357.4	3.47	0.0000	HOMO-4→LUMO+1(+94%)
	9	352.0	3.52	0.5981	HOMO-3→LUMO+1(+57%) HOMO-1→LUMO(13%)
					HOMO→LUMO+1(+7%)
	10	350.0	3.54	0.3016	HOMO-3→LUMO(+61%) HOMO-1→LUMO+1(+9%)
					HOMO-5→LUMO+1(8%) HOMO→LUMO(+5%)
**3**	1	590.3	2.10	0.0457	HOMO→LUMO(+43%) HOMO→LUMO+1(+25%)
					HOMO-1→LUMO+1(20%) HOMO-1→LUMO(+14%)
	2	554.4	2.24	0.0689	HOMO→LUMO+1(+36%) HOMO→LUMO(26%)
					HOMO-1→LUMO(+25%) HOMO-1→LUMO1(+10%)
	3	492.5	2.52	0.0061	HOMO→LUMO+2(+96%)
	4	481.1	2.58	0.0961	HOMO→LUMO+3(+83%) HOMO-1→LUMO+1(+9%)
	5	441.1	2.81	0.1676	HOMO-1→LUMO+3(+44%) HOMO-1→LUMO(+24%)
					HOMO-1→LUMO+2(19%) HOMO→LUMO+1(6%)
	6	438.3	2.83	0.0366	HOMO-1→LUMO+2(+80%) HOMO-1→LUMO+3(+12%)
	7	409.5	3.03	0.0004	HOMO-2→LUMO(+97%)
	8	405.8	3.06	0.1798	HOMO-3→LUMO(+72%) HOMO-1→LUMO+1(10%)
	9	403.0	3.08	0.5780	HOMO-3→LUMO+1(+44%) HOMO-1→LUMO+3(22%)
					HOMO→LUMO+1(6%) HOMO-1→LUMO(+5%)
	10	400.2	3.10	0.0256	HOMO-2→LUMO+1(+92%)
**6**	1	552.3	2.24	0.0469	HOMO→LUMO(+65%) HOMO-1→LUMO+1(36%)
	2	544.0	2.28	0.0100	HOMO→LUMO+1(+54%) HOMO-1→LUMO(+46%)
	3	476.7	2.60	0.0095	HOMO→LUMO+2(+95%)
	4	468.1	2.65	0.1128	HOMO→LUMO+3(+77%) HOMO→LUMO(9%)
					HOMO-1→LUMO+1(8%)
	5	447.4	2.77	0.0164	HOMO-1→LUMO+2(+85%) HOMO-1→LUMO+3(9%)
	6	444.6	2.79	0.0870	HOMO-1→LUMO+3(+58%) HOMO-1→LUMO(18%)
					HOMO-1→LUMO+2(+14%) HOMO→LUMO+1(+6%)
	7	403.3	3.07	0.0039	HOMO-2→LUMO(+96%)
**1**	1	581.4	2.13	0.0245	HOMO→LUMO+1(+61%) HOMO-1→LUMO(30%)
					HOMO→LUMO(+6%)
	2	544.3	2.28	0.0440	HOMO→LUMO(+59%) HOMO-1→LUMO+1(+32%)
					HOMO→LUMO+1(6%)
	3	399.7	3.10	0.9878	HOMO-1→LUMO(+48%) HOMO→LUMO+1(+22%)
					HOMO-7→LUMO+1(+15%)
	4	390.6	3.17	1.1135	HOMO-1→LUMO+1(+43%) HOMO→LUMO(23%)
					HOMO-5→LUMO(+16%)
	5	385.3	3.22	0.0247	HOMO-2→LUMO+1(+55%) HOMO-2→LUMO(39%)
	6	382.8	3.24	0.0034	HOMO-2→LUMO(+51%) HOMO-2→LUMO+1(+34%)
					HOMO-4→LUMO(+13%)
	7	378.8	3.27	0.0145	HOMO-3→LUMO+1(+69%) HOMO-6→LUMO+1(8%)
					HOMO-4→LUMO+1(7%)
**4**	1	540.7	2.29	0.0162	HOMO→LUMO(+57%) HOMO-1→LUMO+1(+38%)
	2	540.5	2.29	0.0157	HOMO→LUMO+1(+57%) HOMO-1→LUMO(39%)
	3	391.9	3.16	1.1281	HOMO-1→LUMO+1(+34%) HOMO→LUMO(23%)
					HOMO-5→LUMO(+12%) HOMO-1→LUMO(+10%)
					HOMO→LUMO+1(+7%) HOMO-3→LUMO(+5%)
	4	390.7	3.17	1.1551	HOMO-1→LUMO(+35%) HOMO→LUMO+1(+25%)
					HOMO-5→LUMO+1(15%) HOMO-1→LUMO+1(11%)
					HOMO→LUMO(+7%)
	5	380.1	3.26	0.0156	HOMO-2→LUMO(+52%) HOMO-2→LUMO+1(43%)
	6	377.4	3.28	0.0002	HOMO-2→LUMO+1(+44%) HOMO-2→LUMO(+36%)
					HOMO-4→LUMO+1(+14%)
	7	372.2	3.33	0.0140	HOMO-3→LUMO(+87%) HOMO-3→LUMO+1(+6%)

It was previously stressed that the frequencies of all asymmetric stretchings of the C_α_-C_m_ bonds, in the ground state of free-base porphin, are very close to each other in the region 1600 cm^-1^ [16,36,40]. This concerns not only *ν*_10_*, ν*_19_, but also the IR active b_2u_ modes in free-base porphyrin; one of them is denoted as ω_92_ = 1640 cm^-1^ in [42]. In our case of compound **2** (Table S2), this is easily identified as the *ν*_233_ mode. Being active in IR absorption, this mode can provide a moderate contribution to the long-wavelength tail of the fluorescence spectrum.

**Figure 8:**
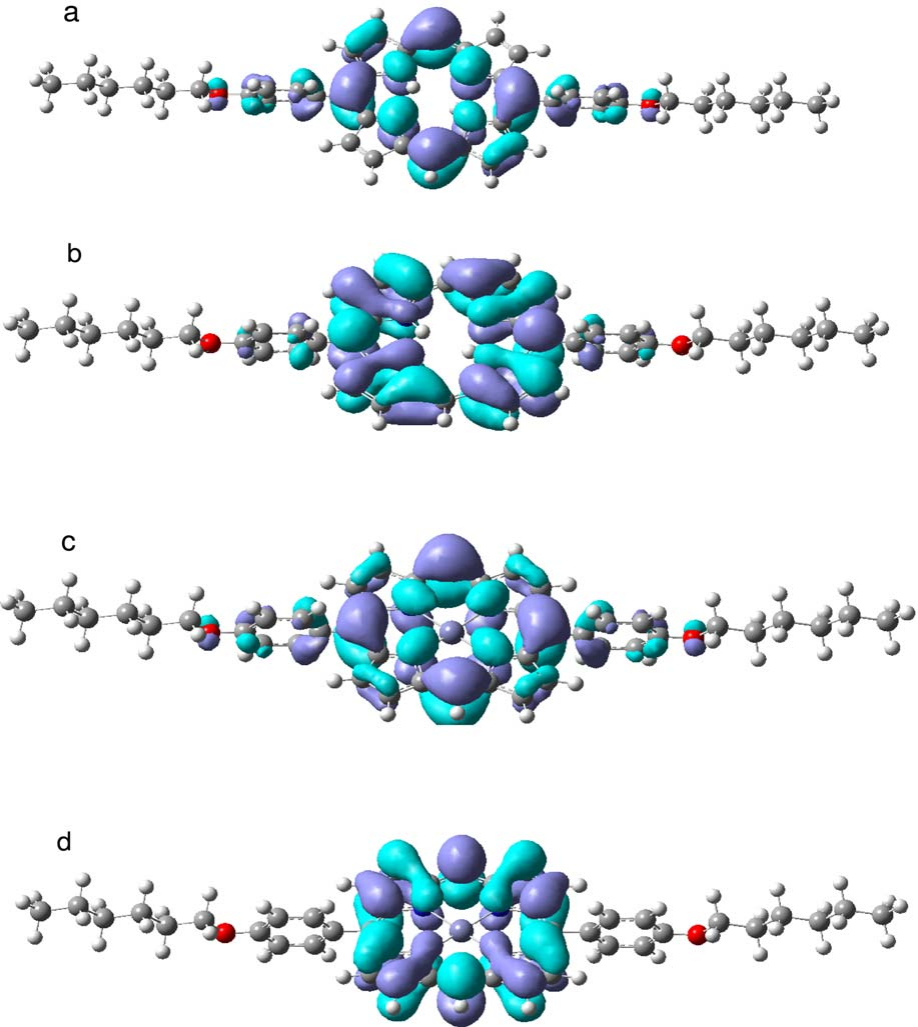
HOMO and LUMO molecular orbitals of compound **2** and **5**: a) HOMO of **2**; b) LUMO of **2**; c) HOMO of **5**; d) LUMO of **5**.

The TD DFT calculations (Table 3, Figure 8) provide additional support for the above mentioned trends in electronic spectra of the studied porphyrins. In the most asymmetric species (compounds **2** and **5**), the presence of only two phenyl rings causes a pronounced hypsochromic shift of all absorption bands in comparison with tetraphenyl derivatives. This trend is well reproduced in our TD DFT calculations (Table 3); even the active HOMO and LUMO orbitals (Figure 8) are quite perturbed, though they are usually pretty well conserved in symmetric porphyrin derivatives [16,25,37,47]. The HOMO orbital in Zn-porphyrin compound **5** (Figure 8c) is a deformed a_2u_ MO. In simple porphyrins the highest occupied orbital is often the a_2u_ MO with a close lying a_1u_ orbital (HOMO-1) [37,47], but in our case the energy gap is larger. This is because the a_2u_ HOMO has considerable contributions from oxyphenyl rings and from the lone pairs at oxygen atoms, but the a_1u_ orbital (HOMO-1) has no admixtures from substituents because it is symmetry forbidden. The LUMO (Figure 8d) is a deformed e_g_ orbital of ZnP, being strongly localized in the porphyrin ring. In the free-base porphin derivative (compound **2**), both orbitals have contributions from oxi-phenyl rings (Figure 8a and 8b). In this case the HOMO is analoguos to the 5b_1u_ orbital of free-base porphyrin (x-axis is along the NH bonds). The LUMO (Figure 8b) is a perturbed variant of the 4b_3g_ orbital of free-base porphyrin. Usually, the LUMO is a 4b_2g_ orbital in symmetric free-bases. An additional reason for pronounced hypsochromic shift (10 to 15 nm) of the Soret (B) bands in the presence of only two phenyl rings in compound **2**, for example, can be explained by a strong admixture of 188–193 (HOMO-3-LUMO+1) excitation, a phenomenon that cannot be predicted within the simple four-level scheme of Gouterman [47].

Comparing porphyrins to Zn-porphyrins, the absorption to the Soret band is shifted to the red and emission is shifted towards the blue side. The red-shift of the Soret absorption is reproduced in all compounds (**1**,**4**,**3**,**6**) behind the tetraphenyl derivatives. The blue-shift of Q bands and hence the blue‑shift of emission on going from porphyrins to Zn-porphyrins is simplified, with only one vertical Q band due to quasi-degeneracy of the B_2u_ and B_1u_ states in Zn-complexes. This is also well reproduced in the calculations (Table 3). In fact, the calculation of compound **5** predicts a small splitting of not only vibronic Q-band, but also of pure electronic Q transitions, corresponding to 1^1^E_u_ states in symmetric ZnTPP molecule of the D_4h_ point group. The calculated Q states splitting is very small (529.5 and 528.4 nm) and could not be resolved, but their band intensities are very different (oscillator strengths are 0.0001 and 0.0164, respectively).

In symmetric ZnTPP, the transitions to 1^1^E_u_ states are forbidden. Thus, disubstitution in porphyrins influence not only Soret bands but also very weak Q band spectra. In tetraphenyl compounds, the structure of Q bands is quite clear. In the case of nitrogroups-containing compounds **3** and **6,** our TD DFT calculations predict a number of weak new transitions of a charge-transfer nature. Predicted absorption, which starts from 440–460 nm (Table 3), we attribute to the broadening of the Soret band (Figure 6). At the same time, the Soret band in these compounds is much weaker, in good agreement with calculations. The shoulder, at 492 nm in the absorption spectrum of compound **3** (Figure 6), we tentatively assign to the weak S_0_–S_3_ transition predicted from the calculations (Table 3).

The disubstitution in compound **5** permits one to see new important weak bands which are usually not seen in other porphyrin and TPP molecules. These are earlier predicted weak σ → π* transitions in the range 350 nm. Here they are detected in the experimental spectrum and also predicted by the TD DFT calculated wavelengths. The second σ → π* transition in the range 300 nm is also connected with 3d-electrons excitation. These singlet ^1^σπ* states are important in the singlet-triplet states mixing induced by spin-orbit coupling on Zn ion, which finally [4] provides a decrease of the phosphorescence radiative lifetime in ZnTPP. The Soret band does not need a special comment, but the left wing seems to be important. Quadratic response TD-DFT method including spin-orbit coupling (SOC) has previously proven to be successful for the phosphorescence lifetime calculations [16]. In this case, the appearance of a new dark ^1^σπ* state, in the vicinity of the Soret band, was predicted and found quite important for SOC analysis and for phosphorescence lifetime calculations. Excitation of Zn-porphyrin in the Soret band leads to a fast internal conversion to the lowest singlet excited state (S_1_); after that about 5% of molecules decay back to the ground state S_0_ emitting fluorescence, while the S_1_ –T_1_ intersysten crossing (ISC) pushes a large part of them (about 90%) to the lowest triplet state (T_1_) [35]. The dark ^1^σπ* state in the vicinity of the Soret band is getting more prominent in absorption spectra of compound **1–6** as a left wing. This can be connected with the observed kinetics of fluorescence in studied porphyrins.

## 3. Methods

### 3.1. Quantum Chemical Calculations

The present calculations of infrared and UV absorption spectra used three model chromophores of asymmetric TPPs (H2 and Zn form) according to Figure 1. The underlying molecular property calculations are performed at the adiabatic DFT level of theory with use of the correlation consistent basis sets of Dunning [20]. Force fields were determined using the hybrid B3LYP exchange‑correlation functional [21] with effective-core potentials (ECP) for Zn, in conjunction with the double‑zeta basis set (cc-pVDZ) for C, N, O and H atoms. The geometry optimization and the calculations of force fields were performed with the Gaussian program [19].

### 3.2. Optical and Luminescence Spectroscopy

UV/Vis absorption measurements were recorded on a JASCO V550 spectrometer. Fluorescence spectra were measured using a Horiba-Jobin Yvon Fluorolog-3 spectrofluorimeter, equipped with a red-sensitive Hamamatsu R928 photomuliplier tube. Spectra were reference corrected for both the excitation source light intensity variation (lamp and grating) and the emission spectral response (detector and grating). Fluorescence quantum yield were measured relative to Coumarin 153 (laser grade, purchased from Acros) in methanol. All solvents were of spectrophotometric grade. Steady state absorption spectra were also recorded using a Shimadzu UV-1601PC Spectrophotometer in conjunction with the luminescence measurements. Spectra were taken with samples diluted to approximately 10–25 μM in tetrahydrofuran (99.5% spectrophotometric grade, from Sigma-Aldrich) using 10 mm quartz cells (Hellma Precision).

Luminescence spectra and excited state lifetimes were recorded using a Jobin Yvon IBH FluoroCube photon-counting spectrometer as the detection unit. The system was equipped with a TBX‑04 picosecond photon detection module for detection in the UV/visible region (300–800 nm) and a Hamamatsu NIR PMT module (H9170–75) for detection in the range 900–1600 nm. A 405 nm Nano‑LED was used as excitation source. Fluorescence emission lifetimes were measured using the luminescence spectrometer in time-correlated single photon counting (TC-SPC) mode. For further details on the set-up, see [41]. Fluorescence spectra were generally recorded at lower concentrations, approximately 0.5–2 μm and assuring no self-absorption or other concentration dependence on the recorded signal.

### 3.3. FTIR Spectroscopy

The transmission infrared spectra of the synthesized compounds were grinded into KBr matrix and formed into pellets. Spectra were recorded employing a VERTEX 70 spectrometer at 5 mbar pressure. A DTGS detector was used and 200 interferograms were averaged at 4 cm^-1^ resolution.

### 3.4. X-Ray Diffraction

Data collection: Processing of the data was performed by the KappaCCD analysis softwares [30]. The lattice constants were refined by least-square refinement. No absorption correction was applied to the data sets [27,28,29].

Structure solution and refinement: Each system was attributed according to the observed systematic extinctions and the structures have been solved in the appropriate space group. The structure was solved by direct methods using the SIR97 program [31] combined with Fourier difference syntheses and refined against *F* using reflections with [*I/σ(I)* > 2] with the CRYSTALS program [32] for all compounds. All atomic displacement parameters for non-hydrogen atoms have been refined with anisotropic terms. After anisotropic refinement, all the hydrogen atoms are found with a Fourier Difference.

### 3.5. NMR Characterization

^1^H and ^13^C NMR spectra were recorded in deuterated chloroform at room temperature on Bruker AC 500 spectrometer. ^13^C NMR spectra were recorded with complete proton decoupling. Chemical shifts are reported in ppm from tetramethylsilane with the solvent resonance as internal standard. For proton, data are reported as follows: chemical shift, integration, multiplicity (s = singlet, d = doublet, t = triplet, q = quartet, m = multiplet, b = broad), coupling constants in Hz. Low and high-resolution mass spectra were performed at the Service Central d’Analyse du CNRS (Vernaison, France).

### 3.6. Synthesis of Compounds

Dipyrromethane [23] and 5-(4-nitrophenyl)dipyrromethane [24] were synthesized from pyrrole and formaldehyde and *para*-nitrobenzaldehyde respectively according to literature procedures. Solvents and reagents were used as purchased. Thin-layer chromatography (TLC) was performed with Merck 60F254 precoated silica gel plates. Column chromatography was carried out using Merck silica gel 60 (70–230 mesh) or basic alumina (grade I) from Merck. DDQ refers to 2,3-dichloro-5,6-dicyano-1,4-benzoquinone, DBU to 1,8-Diazabicyclo[5.4.0]undec-7-ene.

#### 3.6.1. Synthesis of 1: 5,10,15,20-tetra(4-hexyloxyphenyl) porphyrin

A solution of 4-(hexyloxy)benzaldehyde (3 g, 14.5 mmol) and pyrrole (1 mL, 14.5 mmol) in CH_2_Cl_2_ (145 mL) was degassed by bubbling argon for 10–15 min. TFA (1.1 mL) was added via syringe to the solution. The progress of the reaction was monitored by taking aliquots from the reaction mixture and oxidizing with DDQ. After 10 min to 20 min, DDQ (2.468 gr, 10.875 mmol) was added. The mixture was stirred for 1 h and was then filtered directly through a short Alumina column eluting with CH_2_Cl_2_. MeOH was added to eluent to precipitate the porphyrin. After filtration, the product was further purifed by column chromatography on silica eluting with CH_2_Cl_2_. The product was obtained as purple solid. Yield: 405 mg (11%).

ν_max_ (KBr, cm^-1^): 3289 (N-H stretch), 3033, 2950–2854, 1508–1608, 1243, 1106. ^1^H NMR (500 MHz, CDCl_3_): δ(ppm) 8.85 (8H, s, *C_β_-H*), 8.09 (8H, d, *J* = 8.45 Hz), 4.23 (8H, t, *J* = 6.5 Hz, *-OCH_2_-*), 1.96 (8H, m, *-OCH_2_CH_2_-*) 1.61 (m, *-OCH_2_CH_2_CH_2_-*), 1.44 (16H, m), 0.97 (12H, t, *J* = 7 Hz, *-CH_3_*), -2.75 (2H, broad s, NH). ^13^C NMR (125 MHz, CDCl_3_): δ(ppm) 159.9 (*C^IV^*-O), 135.5, 134.4, 119.7, 112.6, 68.3 (-*OCH_2_*-), 31.7 (-*CH_2_*-), 29.4 (-*CH_2_*-), 25.9 (-*CH_2_*-), 22.7 (-*CH_2_*-), 14.1 (-*CH_3_*). MS (ES^+^): *m/z* 1015.6 (M+H)^+^. HRMS (ES^+^): Calcd for C_68_H_79_N_4_O_4_1015.6101, found 1015.6127.

#### 3.6.2. General procedure for the preparation of trans-A_2_B_2_ meso-tetraarylporphyrins

A standard reaction was performed in a 1 L, two-necked, round-bottom flask fitted with a gas inlet port with argon flow maintained for about 5 min. Samples of a stoechiometric amount of dipyrromethane and benzaldehyde were dissolved in undistilled CH_2_Cl_2_ (to obtain 10^-2^ M in reactant). After the solution was purged with argon for 10–15 min, TFA (1.78 eq) was added drop by drop via a syringe over 30 s. Aliquots were oxidized with DDQ to monitor the progress of the reaction by TLC. The reaction mixture was stirred at room temperature until no starting materials remained. DDQ (1.5 eq, 3 eq per porphyrinogen) was then added and the reaction mixture was stirred at room temperature for a further 1 h. During this time, the solution turned from red to dark green. The complete reaction mixture was filtered through a short basic alumina (grade I) column eluting with dichloromethane until the eluted solution was brown. After evaporation of the solvent, the resulting solid was washed with methanol until the filtrate was clean to remove polypyrromethene components.

#### 3.6.3. Synthesis of 2: 5,15-bis(4-hexyloxyphenyl) porphyrin

Condensation of dipyrromethane (500 mg, 3.4 mmol) and 4-(hexyloxy)benzaldehyde (705 mg, 3.4 mmol) in CH_2_Cl_2_ (340 mL) with TFA (470 µl, 6.05 mmol) following the general procedure gave 290 mg of 5,15-bis(4-hexyloxyphenyl)porphyrin (26%).

ν_max_ (KBr, cm^-1^): 3289 (N-H stretch), 3040, 2850–2919, 1502–1604, 1245, 1045. ^1^H NMR (500 MHz, CDCl_3_): δ(ppm) 10.27 (2H, s, *meso H*), 9.37 (4H, d, *J* = 4,35 Hz, *C_β_−H*), 9.09 (4H, d, *J* = 4,35 Hz, *C_β_−H*), 8.15 (4H, d, *J* = 7.95 Hz), 7.3 (4H, d, *J* = 7.95 Hz), 4.26 (4H, t, *J*=6,2 Hz, *-OCH_2_*-), 1.99 (4H, m, *-OCH_2_CH_2_-*), 1.64 (4H, m, -*OCH_2_CH_2_CH_2_-*), 1.46 (8H, m), 0.99 (6H, t, *J* = 6.8 Hz, *-CH_3_*), -3.09 (2H, broad s, -*NH*). ^13^C NMR (125 MHz, CDCl_3_): δ(ppm) 159 (*C*^IV^-O), 135.8, 133.5, 131.4 (*C_β_*), 131.0 (*C_β_*), 118.9, 113, 105.1 (*C meso*), 31.7 (*-*O*CH_2_*-), 29.4 (-*CH_2_*-), 25.9 (-*CH_2_*-), 22.7 (-*CH_2_*-), 14.1 (*-CH_3_*). MS-TOF( ES^+^): *m/z* 663.4 (M+H)^+^. HRMS (ES^+^): Calcd for C_44_H_47_N_4_O_2_ 663.3699, found 663.3703.

#### 3.6.4. Synthesis of 3: 5,15-bis-(4-nitrophenyl)-10,20-bis(4-hexyloxyphenyl) porphyrin

Condensation of 5-(4-nitrophenyl)dipyrromethane (500 mg, 1.87 mmol) and 4-(hexyloxy)benzaldehyde (385 mg, 1.87 mmol) in CH_2_Cl_2_ (190 ml) with TFA (250 µl, 3.32 mmol) following the general procedure gave 140 mg. of 5,15 bis-(4-nitrophenyl)-10,20-bis(4-hexyloxyphenyl)porphyrin (16%).

ν_max_ (KBr, cm^-1^): 3316 (N-H stretch), 3033, 2927–2856, 1599, 1517,1399, 1346, 1286, 1015. ^1^H NMR (500 MHz, CDCl_3_): δ(ppm) 8.92 (4H, d, *J* = 4.1 Hz, *C_β_-H*), 8.72 (4H, d, *J* = 4.1 Hz, *C_β_−H*), 8.62 (4H, d, *J* = 8,55 Hz), 8.37 (4H, d, *J* = 8,55 Hz), 8.07 (4H, d, *J* = 8,55 Hz), 7.27 (4H, d, *J* = 8,5 Hz), 4.23 (4H, t, *J* = 6,5 Hz, *-OCH_2_*-), 1.96 (4H, m, *-OCH_2_CH_2_-*), 1.16 (4H, m, -*OCH_2_CH_2_CH_2_-*), 1.44 (8H, m), 0.96 (6H, t, *J* = 6,6 Hz, *-CH_3_*), -2.78 (1H, broad s, NH), -2.79 (1H, broad s, NH). ^13^C NMR (125 MHz, CDCl_3_): δ(ppm) 159.2 (*C^IV^*-O), 149.0, 147.8 (*C^IV^*-NO_2_), 135.6, 135.1, 133.7 (*C_β_*), 121.9, 121.08, 117.3, 116.8, 112.9, 68.4 (-*CH_2_*-), 31.7 (-*CH_2_*-), 29.4 (-*CH_2_*-), 25.9 (-*CH_2_*-), 22.6 (-*CH_2_*-), 14.0 (*-CH_3_*). Anal. Calcd. for (C_56_H_52_N_6_O_6_) C 74.32, H 5.79, N 9.29; Found: C 74.10, H 5.81, N 9.23. MS (ES^+^): *m/z* 905.4 (M+H)^+^. HRMS (ES^+^): Calcd for C_56_H_53_N_6_O_6_905.4027, found 905.4027.

#### 3.6.5. Preparation of Zn-porphyrins: method A

Free-base porphyrin (1 eq) was dissolved in chloroform and Zn(OAc)_2_.2H_2_O (10 eq) in MeOH was added in one portion. The mixture was stirred vigorously for 6 h at reflux temperature. Reaction progress is monitored by TLC, spotting directly from the reaction mixture. Then, the solvent was removed, and the residue was purified by column chromatography on silica gel eluting with a CHCl_3_/pentane mixture (5/2, v/v). The product corresponds to the first band eluted.

#### 3.6.6. Preparation of Zn-porphyrins: method B

Free-base porphyrin (1eq) was dissolved in CH_2_Cl_2_: THF (1:1) solution containing a 10-fold excess of Zn(OAc)_2_.2H_2_O. Several drops of DBU were added to the solution. The mixture was stirred vigorously for 2h at reflux temperature. Then, the solvent was removed, and the residue was chromatographed on silica gel column with a CHCl_3_/pentane mixture (5/2, v/v). The product corresponds to the first band eluted.

#### 3.6.7. Synthesis of 4: [5,10,15,20-tetra(4-hexyloxyphenyl) porphyrinato] zinc(II)

Starting from 5,10,15,20-Tetra(4-hexyloxyphenyl) porphyrin (50 mg, 0.049 mmol) in 20 ml of CH_2_Cl_2_:THF (1:1), Zn(OAc)_2_.2H_2_O (90 mg, 0.49 mmol), following the general procedure Method B. Yield: 42 mg, 79%.

ν_max_ (KBr, cm^-1^): 3040, 2950–2854, 1509–1608. ^1^H NMR (500 MHz, CDCl_3_): δ(ppm) 8.96 (8H, s, *C_β_−H*), 8.09 (8H, d, *J* = 8.45 Hz), 7.25 (8H, d, *J* = 8.45 Hz), 4.23 (8H, t, *J* = 6.50 Hz, *-OCH_2_*-), 1.97 (8H, m, -*OCH_2_CH_2_CH_2_-*), 1.45 (8H, m, -*OCH_2_CH_2_CH_2_-*), 0.98 (12H, t, *J* = 7.0 Hz, *-CH_3_*). ^13^C NMR (125 MHz, CDCl_3_): δ(ppm) 158.7 (*C^IV^*-O), 150.4 (*C_α_*), 135.3, 135.0, 131.8 (*C_β_*), 120.8, 112.5, 68.3 (-*OCH_2_*-), 31.7 (-*CH_2_*-), 29.4 (-*CH_2_*-), 25.9 (-*CH_2_*-), 22.7 (-*CH_2_*-), 14.1 (*-CH_3_*). MS( ES^+^): *m/z* 1077.6 (M+H)^+^. HRMS (ES^+^): Calcd for C_68_H_76_N_4_O_4_Zn 1076.5158, found 1076.5122.

#### 3.6.8. Synthesis of 5: [5,15-bis(4-hexyloxyphenyl) porphyrinato] zinc(II)

Starting from 5,15-bis(4-hexyloxyphenyl)porphyrin (50 mg, 0.07 mmol) in CHCl_3_(20 mL), Zn(OAc)_2_.2H_2_O (165 mg, 0.57 mmol) in MeOH (5 mL) following the general procedure Method A. 22 mg, 38% yield.

ν_max_ (KBr, cm^-1^): 3034, 2946–2850, 1500–1604, 1241, 1056. ^1^H NMR (500 MHz, CDCl_3_): δ(ppm) 10.29 (2H, s, meso H), 9.41 (4H, d, *J* = 4.45 Hz, *C_β_−H*), 9.16 (4H, d, *J* = 4.45 Hz, *C_β_−H*), 8.14 (4H, d, *J* = 8.45 Hz), 7.30 (4H, d, *J* = 8.45 Hz), 4.27 (t, *J* = 6.50 Hz, -*OCH_2_-*), 2.00 (4H, m, -*OCH_2_CH_2_-*), 1.65 (4H, m, -*OCH_2_CH_2_CH_2_-*), 1.46 (8H, m), 0.99 (6H, t, *J* = 7 Hz, *-CH_3_*). ^13^C NMR (125 MHz, CDCl_3_): δ(ppm) 158.9 (*C^IV^*-O), 150.5 (*C_α_*), 149.4 (*C_α_*), 135.6, 134.7, 132.5 (*C_β_*), 131.6 (*C_β_*), 112.7, 106.1 (*C meso*), 68.4, 31.7, 29.7, 29.5, 25.9, 22.7, 14.1 (*-CH_3_*). MS (ES^+^): *m/z* 725.3 (M+H)^+^. HRMS (ES^+^): Calcd for C_44_H_45_N_4_O_2_Zn 725.2822, found 725.2834.

#### 3.6.9. Synthesis of 6: [5,15-bis-(4-nitrophenyl)-10,20-bis(4-hexyloxyphenyl) porphyrinato] zinc(II)

Starting from 5,15 bis-(4-nitrophenyl)-10,20-bis(4-hexyloxyphenyl)porphyrin (50 mg, 0.055 mmol) in CHCl_3_ (20 ml), Zn(OAc)_2_.2H_2_O (120 mg, 0.55 mmol) in MeOH (5 ml) following the general procedure Method A. 22 mg, 41% yield.

ν_max_ (KBr, cm^-1^): 3033, 2948–2850, 1596, 1521, 1384,1340, 1284,1072. ^1^H NMR (500 MHz, CDCl_3_): δ(ppm) 9.02 (4H, d, *J* = 4.65 Hz, *C_β_−H*), 8.81 (4H, d, *J* = 4.65 Hz, *C_β_−H*), 8.61 (4H, d, *J* = 8.55 Hz), 8.37 (4H, d, *J* = 8.5 Hz), 8.07 (4H, d, *J* = 8.45 Hz), 7.26 (4H, d, *J* = 8.45 Hz), 4.23 (t, *J* = 6.50 Hz, -*OCH_2_-*), 1.96 (4H, m, -*OCH_2_CH_2_-*), 1.60 (4H, m, -*OCH_2_CH_2_CH_2_-*), 1.44 (8H, m), 0.96 (6H, t, *J* = 7 Hz, *-CH_3_*). ^13^C NMR (125 MHz, CDCl_3_): δ(ppm) 159.0 (*C^IV^*-O), 150.9 (*C_α_*), 149.8, 149.2 (*C_α_*), 147.6 (*C^IV^*-NO_2_), 135.4, 134.9, 134.39, 132.9 (*C_β_*), 131.2 (*C_β_*), 121.9, 121.75, 118.8, 112.73, 68.3 (-*OCH_2_*-), 31.7 (-*CH_2_*-), 29.4 (-*CH_2_*-), 25.9 (-*CH_2_*-), 22.6 (-*CH_2_*-), 14.0 (-*CH_3_*). MS(ES^+^): *m/z* 967.3 (M+H)^+^. HRMS (ES^+^): Calcd for C_56_H_51_N_6_O_6_Zn 967.3163, found 967.3162.

## 4. Conclusions

The synthesis procedure of a series of new asymmetrically substituted free-base di- and tetra-phenylporphyrins and their corresponding Zn-phenylporphyrins were described along with detailed studies of their structural, vibrational and electronic properties. Specifically, X-ray diffraction and NMR showed essentially planar ring structures and the corresponding infrared and electronic absorption spectra showed small differences from the results of symmetrically substituted tetraphenyl porphyrins described in the literature. The small differences in FTIR and fluorescence were analyzed by means of detailed density functional theory (DFT) calculations. All calculated vibrational modes (2162 modes for all six compounds studied) were assigned to the observed FTIR spectra. Absorption spectra in UV and the visible regions of all compounds show the typical *ethio* type feature of *meso*‑tetraarylporphyrins with a very intense Soret band and weak Q bands of decreasing intensity. In diphenyl derivatives, the presence of only two phenyl rings causes a pronounced hypsochromic shift of all bands in the absorption spectra. Time-dependent DFT calculations revealed some peculiarities in the electronic excited states structure and connected them with vibronic bands in absorption and fluorescence spectra from associated vibrational sublevels. The fluorescence emissions showed similar features to symmetrically substituted analogues with decay times in the range 4–5 ns for the proto‑form, shortened to approx 1–2 ns for the Zn-substituted counterparts.

## Supporting information

Table S1: Bond distances and angles from crystal structure of compounds **4**–**6**.

Table S2–S4: Calculated IR frequencies of studied di- end tetra-phenyl porphyrins.
