# Supplementary material for: Fluorescence and FTIR Spectra Analysis of Trans-A2B2-Substituted Di- and Tetra-Phenyl Porphyrins
Source: Materials (Basel). 2010 Aug 23;3(8):4446–75. doi: 10.3390/ma3084446 (PMC5445833; doi:10.3390/ma3084446)
Supplement: Supplementary file 1 [file materials-03-04446-s001.doc]

**Table S1.** Selected Bond Distances (Å) and Angles (°) in Complexes **4**, **5** and **6**.

|  | Zn–N | | N–Zn–N | | N–C_α_ | | C_α_–C_β_ | | C_α_–C_meso_ | | C_β_–C_β_ | |
| --- | --- | --- | --- | --- | --- | --- | --- | --- | --- | --- | --- | --- |
| **4** | Zn1–N3 | 2.045 (2) | N3–Zn1–N4 | 89.97 (10) | N3–C16 | 1.374 (4) | C5–C18 | 1.434 (4) | C10–C6 | 1.403 (5) | C15–C20 | 1.347 (5) |
|  | Zn1–N4 | 2.026 (2) | N4^#^–Zn1–N3 | 90.03 (10) | N3–C5 | 1.376 (4) | C10–C20 | 1.432 (5) | C16–C6 | 1.394 (5) |  |  |
|  |  |  |  |  | N4–C20 | 1.379 (4) | C16–C24 | 1.437 (4) | C5–C11 | 1.406 (4) | C18–C24 | 1.347 (5) |
|  |  |  |  |  | N4–C22 | 1.369 (4) | C22–C15 | 1.448 (4) | C22–C11 | 1.395 (4) |  |  |
| **5** | Zn1–N2 | 2.021 (7) | N2–Zn1–N6 | 89.5 (3) | N2–C3 | 1.378 (11) | C3–C13 | 1.430 (12) | C3–C4 | 1.398 (13) | C8–C9 | 1.335 (13) |
|  | Zn1–N6 | 2.022 (8) | N2*–Zn1–N6 | 90.5 (3) | N2–C11 | 1.378 (12) | C5–C9 | 1.411 (13) | C5–C4 | 1.400 (12) |  |  |
|  |  |  |  |  | N6–C5 | 1.388 (11) | C7–C8 | 1.433 (14) | C11–C10 | 1.361 (14) | C12–C13 | 1.332 (14) |
|  |  |  |  |  | N6–C7 | 1.371 (12) | C11–C12 | 1.419 (13) | C7–C10 | 1.379 (13) |  |  |
| **6** | Zn1–N2 | 2.031 (7) | N2–Zn1–N24 | 90.3 (3) | N2–C4 | 1.394 (11) | C4–C34 | 1.438 (13) | C4–C6 | 1.409 (12) | C28–C29 | 1.323 (13) |
|  | Zn1–N24 | 2.025 (7) | N24&–Zn1–N2 | 89.7 (3) | N2–C32 | 1.365 (11) | C23–C29 | 1.436 (13) | C23–C6 | 1.416 (13) |  |  |
|  |  |  |  |  | C23–N24 | 1.370 (12) | C27–C28 | 1.454 (13) | C27–C31 | 1.395 (12) | C33–C34 | 1.346 (13) |
|  |  |  |  |  | N24–C27 | 1.379 (10) | C32–C33 | 1.456 (12) | C32–C31 | 1.399 (13) |  |  |
|  | Zn49–N50 | 2.039 (7) | N50–Zn49–N70 | 89.9 (3) | N50—C51 | 1.372 (11) | C51–C75 | 1.445 (12) | C51–C52 | 1.388 (12) | C74–C75 | 1.347 (14) |
|  | Zn49–N70 | 2.050 (7) | N50^&^–Zn49–N70 | 90.1 (3) | N50–C73 | 1.389 (11) | C69–C92 | 1.422 (13) | C52–C69 | 1.408 (12) |  |  |
|  |  |  |  |  | C69–N70 | 1.376 (11) | C71–C91 | 1.467 (12) | C71–C72 | 1.403 (12) | C91–C92 | 1.341 (14) |
|  |  |  |  |  | N70–C71 | 1.344 (11) | C73–C74 | 1.450 (13) | C73–C72 | 1.377 (13) |  |  |

Symmetry transformations used to generate equivalent atoms: ^#^ −*x*, −*y*+1, −*z*+1. * −*x*, −*y*+2, −*z*+1. ^&^ −*x*−1, −*y*+1, −*z*−1.

**Table S2.** Calculated and experimental wavenumbers (*ν,* cm^–1^) and IR absorption intensity (*I*, km/mole) of the normal vibrations of the 10, 20-*bis*(4-hexoxyphenyl)-porphine and of the complex 10, 20-*bis*(4-hexoxyphenyl)-porphine with Zinc in the 1650–600 cm^–1^ range.

| Compound | **2** (DPP) | | | | **5** (ZnDPP) | | | |
| --- | --- | --- | --- | --- | --- | --- | --- | --- |
| Assignments (*cursive in parenthesis corresponds to ZnDPP*) | Mode | *ν_calc._* | *ν_exp._* | *I*_abs_. | Mode | *ν_calc._* | *ν_exp._* | *I*_abs_. |
| *ν_s_*(C=C)_benz_ – out-of-phase/δ(CH)_ph_ | 235 | 1618 | 1605 | 298.6 | 234 | 1619 | 1605 | 259.4 |
| *ν_as_*(C*_m_*–C_α_) | 234 | 1608 |  | 0.5 | 233 | 1606 |  | 0.6 |
| *ν_as_*(C*_m_*–C_α_)/*ν*(C_β_–C_β_) –**II**, **IV**_pyr. ring_ – out-of-phase, *ν_as_*(C_α_–C_β_) – **I**, **III**_pyr. ring_, δ(C*_m_*H)/δ(NH)/ δ(∠C_α_NC_α_) – **II**, **IV**_pyr. ring_ **–** out-of-phase (*ZnDPP – and* δ(∠C_α_NC_α_) – **I**, **III**_pyr. ring_ – out-of-phase) | 233 | 1586 | 1578 | 50.5 | 230 | 1559 | 1553 | 27.9 |
| *ν_s_*(C=C)_benz_ – in phase/δ(CH)_ph_ | 232 | 1572 |  | 38.1 | 232 | 1573 | 1572 | 35.8 |
| *ν*(C*_m_*–С_α_)/*ν*(C_β_–C_β_)/*ν_s_*(С_α_–N)/δ(∠C_α_NC_α_) – **II**, **IV**_pyr. ring_ –out-of-phase, *ν*(C*_m_*–C_benz_) – out-of-phase/δ(C_β_H) – **II**, **IV**_pyr. ring_ (*ZnDPP – and* *ν*(C*_m_* –С_α_)/*ν*(C_β_–C_β_)/*ν_s_*(С_α_–N)/δ(∠C_α_NC_α_) – **I, III**) | 228 | 1545 | 1535 | 17.3 | 228 | 1532 |  | 14.9 |
| *ν_s_*(C*_m_*–C_α_)/*ν*(C_β_–C_β_)/*ν_s_*(С_α_–N) – **I**, **III**_pyr. ring_ **–**out-of-phase/δ(C_β_H) – **I**, **III**_pyr. ring_/*ν_as_*(С_α_–N) – **II**, **IV**_pyr. ring_/*ν*(C*_m_*–C_benz_) – out-of-phase (*ZnDPP – ν*(C*_m_*–C_benz_) – out-of-phase disappear) | 227 | 1518 |  | 24.8 | 227 | 1525 | 1522 | 31.2 |
| *ν*(C=C)_benz_ – out-of-phase/δ(CH)_ph_ | 225 | 1503 | 1505 | 258.3 | 223 | 1501 | 1504 | 369.6 |
| δ(NH)/δ(C_β_H)/δ(CH)/*ν_as_*(C*_m_* –С_α_)/*ν*(C_β_–C_β_) – **I**, **III**_pyr. ring_ – out-of-phase (*ZnDPP –* *and* *ν*(C_β_–C_β_) – **II**, **IV**_pyr. ring_ **–** out-of-phase) | 223 | 1494 |  | 164.9 | 224 | 1502 |  | 3.1 |
| δ(b∠CH_2_) | 219 | 1459 | 1466 | 81.5 | 219 | 1459 | 1466 | 75.4 |
| δ*_as_*(CH_3_)/δ(b∠CH_2_) | 218 | 1449 |  | 8.2 | 218 | 1449 | 1452 | 5.6 |
| δ*_as_*(CH_3_)/δ(b∠CH_2_) | 217 | 1449 |  | 4.8 | 217 | 1449 |  | 3.9 |
| (*ZnDPP – ν*(C_β_–C_β_) – **II**, **IV**_pyr. ring_ – out-of-phase and **I**, **III**_pyr. ring_ – out-of-phase/*ν_as_*(С_α_–N)/δ(C_β_H)/ *ν*(C*_m_*–C_benz_) – out-of-phase) |  |  |  |  | 212 | 1436 | 1429 | 62.3 |
| δ(NH)/δ(C*_m_*H)/*ν*(С_α_–N) | 205 | 1419 | 1418 | 135.2 |  |  |  |  |
| *w*(CH_2_) (*ZnDPP* – *and* *ν*(C_α_– C_β_), δ(C*_m_*H)) | 199 | 1384 | 1387 | 87.7 | 199 | 1384 | 1393 | 146.8 |
| *ν*(C_α_ –C_β_)/*ν*(C_α_ –N)/δ(CH)/ring deformations – **I**, **III**_pyr. ring_/δ(NH) (*ZnDPP* – *and* **II***,* **IV**_pyr. ring_) | 193 | 1357 | 1362 | 13.4 | 192 | 1351 |  | 0.1 |
| *w*(CH_2_)/δ_s_(CH_3_) | 190 | 1338 | 1342 | 23.1 | 190 | 1339 |  | 25.1 |
| *ν*(C=C) – **I**–**IV**_pyr. ring_/δ(∠C_α_NC_α_) | 189 | 1335 |  | 27.9 | 188 | 1322 |  | 10.1 |
| δ(C_β_H) | 188 | 1315 | 1321 | 18.1 | 189 | 1324 |  | 2.6 |
| *ν_as_*(C=C) – **A**, **B**_benz. ring_/*ν_as_*(C_α_–N) – **I**–**IV**_pyr. ring_/δ(NH)/δ(C_β_H) | 187 | 1311 | 1290 | 97.5 | 187 | 1311 | 1315 | 161.2 |
| *ν_as_* (C=C) – **A**, **B**_benz. ring_ | 186 | 1305 |  | 35.3 | 185 | 1304 |  | 9.0 |
| *ν_as_* (C=C) – **A**, **B**_benz. ring_**/**τ(CH)_ph_ /τ(NH) | 185 | 1297 |  | 70.9 | 186 | 1305 |  | 66.1 |
| *w*(CH_2_) – **B**-hexyl/τ(CH)_ph_ – **B**_benz. ring_/*ν*(C*_m_*–C_benz_) – out-of-phase | 180 | 1283 | 1271 | 62.7 | 179 | 1281 | 1281 | 110.8 |
| τ(CH_2_) – **A**-hexyl/τ(CH)_ph_ – **A**_benz. ring_/*ν*(C*_m_*–C_benz_) – out-of-phase | 179 | 1281 |  | 26.3 | 180 | 1284 |  | 25.0 |
| δ(C_β_H) – **I**, **III**_pyr. ring_/δ(C*_m_*H) | 173 | 1261 |  | 5.6 | 169 | 1246 |  | 103.6 |
| *ν*(C_benz_–O) – in phase/*ν*($\text{C}_{m}^{\alpha}$–C_benz_)/*ν*($\text{C}_{m}^{\gamma}$–C_benz_) – in phase/*ν*(C^1^–N)/ *ν*(C^11^–N) – in phase, δ(NH) (*ZnDPP* – *and* *ν*(C^7^–N)/*ν*(C^18^–N) – in phase) | 172 | 1256 |  | 80.4 | 170 | 1249 |  | 8.3 |
|  |  |  |  |  | 172 | 1254 |  | 11.6 |
| *ν_as_*(C_benz_ **_B_**–O–C)/τ(CH)/*ν*(C^1^–N)/*ν*(C^11^–N) – in phase/δ($\text{C}_{m}^{\delta}$H) (*ZnDPP* – *and ν*(C^7^–N)/*ν*(C^18^–N) – in phase) | 171 | 1252 | 1246 | 426.1 | 173 | 1255 | 1244 | 397.9 |
| *ν_as_*(C_benz_ **_A_**–O–C)/τ(CH)/*ν*(C^1^–N)/*ν*(C^11^–N) – in phase/δ($\text{C}_{m}^{\beta}$H) (*ZnDPP* – *and ν*(C^7^–N)/*ν*(C^18^–N) – in phase) | 170 | 1251 |  | 737.9 | 171 | 1250 |  | 751.7 |
| δ(C_β_H) (*ZnDPP* – *and ν*(C^1^–N)/*ν*(C^18^–N) and *ν*(C^7^–N)/*ν*(C^11^–N) – out-of-phase/δ(CH)) | 167 | 1226 |  | 34.9 | 166 | 1227 |  | 2.3 |
| *w*(CH_2_) – **B**-hexyl | 166 | 1215 |  | 12.1 | 165 | 1216 |  | 3.4 |
| *w*(CH_2_) – **A**-hexyl | 165 | 1215 |  | 25.5 | 164 | 1216 |  | 35.3 |
| δ(NH)/δ(C_β_H) | 164 | 1204 |  | 0.02 | 163 | 1208 |  | 18.0 |
| δ(NH) | 163 | 1199 | 1198 | 14.2 |  |  |  |  |
| δ(CH)_ph_ – **A**, **B**_benz. ring_ – out-of-phase | 157 | 1156 | 1175 | 246.2 | 157 | 1156 | 1173 | 218.4 |
| *ν*(C_α_ –N) – **I**–**IV**_pyr. ring_/δ(C_β_H)/δ(C*_m_*H) | 154 | 1140 | 1146 | 27.6 | 154 | 1135 | 1146 | 16.6 |
| *ν*(C–С) – **A**-hexyl (*ZnDPP* – *and* *ν*(C–С) – **B**-hexyl) | 151 | 1115 |  | 19.8 | 152 | 1115 |  | 20.9 |
| δ(CH)_ph_ – **B**_benz. ring_ – out-of-phase (*ZnDPP* – *and* δ(CH)_ph_ – **A**_benz. ring_ – out-of-phase) | 150 | 1096 | 1105 | 11.2 | 151 | 1095 | 1103 | 18.6 |
| δ(CH)_ph_ – **A**_benz. ring_ – out-of-phase (*ZnDPP* – *and* δ(CH)_ph_ – **B**_benz. ring_ – out-of-phase) | 149 | 1095 |  | 15.7 | 150 | 1095 |  | 7.1 |
| *ν_as_*(O–C_hex_ **_B_** –С)/*ν*(C–С) – **B**-hexyl (*ZnDPP* – *and ν*(O–C_hex_ **_A_**–С)/*ν*(C–С) – **A**-hexyl) | 146 | 1048 | 1047 | 23.0 | 147 | 1049 |  | 10.8 |
| *ν*(O–C_hex_ **_A_**–С)/*ν*(C–С) – **A**-hexyl (*ZnDPP* – *and ν_as_*(O–C _hex_ **_B_** –С)/*ν*(C–С) – **B**-hexyl) | 145 | 1048 |  | 61.5 | 146 | 1048 |  | 69.6 |
| δ(C_β_H) – **II**, **IV**_pyr. ring_ – out-of-phase (*ZnDPP* – *and* δ(C_β_H) – **I**, **III**_pyr. ring_ – out-of-phase) | 142 | 1038 |  | 24.0 | 143 | 1041 |  | 9.0 |
| δ(C_β_H) – **I**, **III**_pyr. ring_ – out-of-phase (*ZnDPP* – *and* δ(C_β_H) – **II**, **IV**_pyr. ring_ – out-of-phase) | 141 | 1034 |  | 73.6 | 141 | 1039 | 1059 | 61.1 |
| *ν*(C_hex_–O)/*ν*(C–C) – **A**-hexyl (*ZnDPP* – *and ν*(C_hex_–O)/*ν*(C–C) – **B**-hexyl – in phase) | 140 | 1033 |  | 34.5 | 139 | 1031 |  | 20.5 |
| *ν*(C_hex_ **_A_**–O) and *ν*(C_hex_ **_B_**–O) – out-of-phase/δ(C_β_H) – **I**, **III**_pyr. ring_ **–** out-of-phase (*ZnDPP* – δ(C_β_H) – disappear) | 139 | 1032 |  | 78.2 | 140 | 1040 | 1059 | 113.6 |
| *ν_s_*(C_α_–C_β_) – **I**, **III**_pyr. ring_ – out-of-phase/*ν_as_*(C_α_–C_β_) – **II**, **IV**_pyr. ring_/τ(CH)_ph_ (*ZnDPP* – and *ν_s_*(C_α_–C_β_) – **II**, **IV**_pyr. ring_ – out-of-phase. *ν_as_*(C_α_–C_β_) – **II**, **IV**_pyr. ring_ – disappear) | 133 | 990 | 1018 | 8.9 | 133 | 1001 | 1017 | 27.2 |
| *ν_s_*(C_α_–C_β_) – **I**, **III**_pyr. ring_ – in phase/*ν_as_*(C_α_–C_β_) – **II**, **IV**_pyr. ring_/τ(CH)_ph_ (*ZnHPP* – and *ν_s_*(C_α_–C_β_) –**II**, **IV**_pyr. ring_ **–** in phase. *ν_as_*(C_α_–C_β_) – **II**, **IV**_pyr. ring_ – disappear) | 132 | 989 |  | 3.2 | 132 | 999 |  | 0.3 |
| *ν*(C–C) – **B**-hexyl/*ν*(C_hex_ **_B_**–O)/**B** – ring deformation/τ(C_β_H) (*ZnDPP* – and *ν*(C–C) – **A**-hexyl/*ν*(C_hex_ **_A_**–O)/**A** – ring deformation) | 130 | 989 |  | 2.7 | 129 | 989 |  | 36.7 |
| δ(NH)/τ(C_β_H) – **I**, **III**_pyr. ring_/**A**, **B** – ring deformation | 129 | 985 | 990 | 47.4 |  |  |  |  |
| δ(NH)/τ(C_β_H)/**I**, **II** and **III**, **IV**_pyr. ring_ – breathing – out-of-phase | 126 | 969 | 974 | 52.3 | 126 | 989 | 995 | 140.6 |
| δ(NH)/τ(C_β_H) – **I**, **III**_pyr. ring_/**I**, **III** – ring deformation/**II**, **IV** – breathing (*ZnDPP* – **I**, **IV** and **II**, **III** – breathing – out-of-phase/*ν*(C–C) – **A**, **B**-hexyl) | 125 | 967 |  | 0.2 | 127 | 989 |  | 47.5 |
| **II**, **III**_pyr. ring_ – ring deformation – in phase/γ(CH)_ph_ – **A**, **B**_benz. ring_ | 122 | 955 | 957 | 23.4 |  |  |  |  |
| **I**, **IV**_pyr. ring_ – ring deformation – in phase/γ(CH)_ph_ – **A**, **B** _benz. ring_ | 121 | 954 |  | 34.3 |  |  |  |  |
| γ(CH)_ph_ – **A**_benz. ring_ (*ZnDPP* – *and* γ(CH)_ph_ – **A**, **B**_benz. ring_) | 120 | 937 | 937 | 3.5 | 121 | 938 | 936 | 0.8 |
| γ(CH)_ph_ – **B**_benz. ring_ (*ZnDPP* – *and* γ(CH)_ph_ – **A**, **B**_benz. ring_) | 119 | 936 |  | 2.4 | 120 | 937 |  | 0.7 |
| γ(C_β_H) | 118 | 905 | 903 | 1.4 | 119 | 906 | 909 | 2.0 |
| *τ*(CH_2_) – **B**-hexyl | 114 | 881 | 876 | 1.4 | 115 | 881 |  | 1.6 |
| *τ*(CH_2_) – **A**-hexyl | 113 | 881 |  | 1.4 | 114 | 881 |  | 1.0 |
| *ν*(C–С)/*τ*(CH_3_) – **B**-hexyl | 112 | 879 |  | 1.7 | 113 | 879 |  | 2.3 |
| *ν*(C–С)/*τ*(CH_3_) – **A**-hexyl | 111 | 879 |  | 1.6 | 112 | 879 |  | 0.9 |
| γ(C*_m_*H)/γ(C_β_H) | 108 | 852 | 849 | 58.2 | 109 | 855 | 858 | 51.0 |
| γ(CH)_ph_ – **A**, **B**-ring – in phase | 105 | 844 |  | 19.4 | 107 | 849 |  | 13.6 |
| γ(CH)_ph_ – **B**-ring/**B** – ring deformation (*ZnDPP* – *and* γ(CH)_ph_ – **A**-ring/**A** – ring deformation) | 101 | 811 |  | 10.5 | 102 | 812 |  | 9.9 |
| γ(CH)_ph_ – **A**, **B**-ring – in phase/**I**–**IV**_pyr. ring_ – deformation/*τ*(CH_2_) | 99 | 798 |  | 18.3 | 100 | 804 | 806 | 36.6 |
| γ(NH)/γ(C_β_H)/γ(C*_m_*H) (*ZnDPP* – *and τ*(CH)_2_/*τ*(CH)_3_; γ(C*_m_*H) – disappear) | 98 | 795 | 799 | 86.9 | 99 | 794 |  | 0.2 |
| γ(C_β_H)/γ(C*_m_*H)/γ(NH) (*ZnDPP* – *and τ*(CH)_2_/*τ*(CH)_3_; γ(C*_m_*H) – disappear) | 97 | 794 |  | 9.4 | 98 | 794 |  | 15.0 |
| γ(NH)/γ(CH) – **A**-ring/*τ*(CH)_2_ –**A**-hexyl/γ(C_β_H) | 95 | 792 |  | 13.9 |  |  |  |  |
| γ(C_β_H) | 94 | 782 |  | 8.0 | 96 | 786 |  | 0.2 |
|  |  |  |  |  | 95 | 785 | 789 | 36.9 |
| **I**, **II** and **III**, **IV**_pyr. ring_ – ring deformation – out-of-phase | 89 | 755 |  | 16.5 | 90 | 766 | 772 | 35.9 |
| γ(NH) in phase | 88 | 747 | 745 | 36.3 |  |  |  |  |
| γ(C_β_H)/γ(C*_m_*H) | 84 | 727 |  | 0.5 | 86 | 735 | 737 | 16.9 |
| γ(CH)_ph_/γ(C_benz_) | 80 | 711 | 714 | 7.3 | 82 | 715 | 716 | 5.2 |
| macrocycle – out-of-plane | 78 | 696 | 691 | 13.9 | 81 | 704 | 700 | 13.7 |
| **A**, **B** _benz. ring_ *–* ring deformation | 73 | 633 | 640 | 10.3 | 75 | 635 |  | 9.7 |
| **A**, **B**_benz. ring_ *–* ring deformation/δ(*b*∠C_benz_OC) – out-of-phase | 71 | 620 | 629 | 62.6 | 73 | 621 | 629 | 63.6 |
| γ(CH)_ph_/γ(C_benz_–C*_m_*) | 67 | 553 | 555 | 5.6 | 70 | 556 | 557 | 4.6 |

* *ν –* stretching vibration; deformation vibrations: δ(*b*∠) – scissoring, *w –* wagging*,* τ – twisting; γ – out-of-plane vibrations.

**Table S3.** Calculated and experimental wavenumbers (*ν,* cm^–1^) and IR absorption intensity (*I*, km/mole) of the normal vibrations of the 10,20-*bis*(4-hexoxyphenyl)-5,15-*bis*(4-nitrophenyl)porphine and of the complex 10,20-*bis*(4-hexoxyphenyl)-5,15-*bis*(4-nitrophenyl) with Zinc in the 1650–600 cm^–1^ range.

| Compound | **3** (TPP) | | | | **6** (ZnTPP) | | | |
| --- | --- | --- | --- | --- | --- | --- | --- | --- |
| Assignments (*cursive in parenthesis corresponds to ZnTPP*) | Mode | *ν_calc._* | *ν_exp._* | *I*_abs_. | Mode | *ν_calc._* | *ν_exp._* | *I*_abs_. |
| *ν_s_*(C=C) – **D**_benz. ring_ /*ν_as_*(NO_2_) | 302 | 1624 | 1607 | 113.4 | 301 | 1625 | 1605 | 112.6 |
| *ν_s_*(C=C) – **C**_benz. ring_ /*ν_as_*(NO_2_) | 301 | 1623 |  | 120.0 | 300 | 1624 |  | 120.7 |
| *ν_s_*(C=C) – **B**_benz. ring_ (*ZnTPP – and* **A**_benz. ring_) | 300 | 1619 |  | 87.6 | 299 | 1619 |  | 5.3 |
| *ν_s_*(C=C) – **A**_benz. ring_ (*ZnTPP – and* **B**_benz. ring_) | 299 | 1618 |  | 232.3 | 298 | 1619 |  | 295.8 |
| *ν_s_*(C=C) – **C, D**_benz. ring_ – in phase/δ(CH)_ph_ | 298 | 1602 | 1597 | 12.1 | 297 | 1602 | 1595 | 23.4 |
| *ν_s_*(C=C) – **C, D**_benz. ring_ – out-of-phase/δ(CH)_ph_ | 297 | 1602 |  | 144.6 | 296 | 1602 |  | 131.7 |
| *ν_s_*(C=C) – **A, B**_benz. ring_ – out-of-phase/δ(CH)_ph_ | 296 | 1573 | 1560 | 41.9 | 295 | 1573 |  | 42.3 |
| *ν_s_*(C=C) – **D**_benz. ring_ /*ν_as_*(NO_2_) | 294 | 1570 |  | 51.9 | 293 | 1570 |  | 48.4 |
| *ν_s_*(C=C) – **C**_benz. ring_ /*ν_as_*(NO_2_) | 293 | 1569 |  | 95.1 | 292 | 1569 | 1572 | 115.9 |
| *ν_as_*(C*_m_*–C_α_)/*ν*(C_β_–C_β_) –**II**, **IV**_pyr. ring_ – out-of-phase/*ν_as_*(C_α_–C_β_), *ν_as_*(С_α_–N) – **I**, **III**_pyr. ring_ /δ(NH) | 292 | 1564 |  | 93.3 | – | – | – | – |
| *ν_s_*(C*_m_*–C_α_)/*ν*(C_β_–C_β_)/*ν_s_*(С_α_–N) – **I**, **III**_pyr. ring_ – out-of-phase/δ(C_β_H) – **I**, **III**_pyr. ring_ | 287 | 1519 | 1520 | 13.4 | 289  288 | 1535  1535 | 1522 | 26.9  24.8 |
| *ν_as_*(C=C)_benz. ring_ – out-of-phase/δ(CH)_ph_ | 285 | 1503 |  | 321.2 | 287  286 | 1507  1507 |  | 122.5  58.7 |
| **I**, **II** and **III, IV**_pyr. ring_ – breathing, out-of-phase | 282 | 1481 | 1472 | 119.7 | 282 | 1490 | 1491 | 252.3 |
| *ν_as_*(C=C) – **C, D**_benz. ring_ /δ(CH)_ph_ | 280 | 1474 |  | 71.8 | 280 | 1477 |  | 42.4 |
| δ(b∠CH_2_) **– A**, **B**-hexyl | 278 | 1458 |  | 103.6 | 278  277 | 1457  1457 | 1466 | 22.6  71.8 |
| (*ZnTPP – ν*_as_(C_α_–C_β_) – **I**–**IV**_pyr. ring_) |  |  |  |  | 271 | 1434 | 1433 | 17.7 |
| *ν*_as_(C=C) – **B**_benz. ring_ /δ(CH)_ph_ (*ZnTPP – and* **A**) | 262 | 1405 | 1400 | 10.4 | 260 | 1405 |  | 9.7 |
| *ν*_as_(C=C) – **D**_benz. ring_ /δ(CH)_ph_ | 260 | 1391 |  | 18.4 | 259 | 1391 |  | 10.5 |
| *w*(CH_2_) – **A**-hexyl | 258 | 1385 |  | 32.2 | 257 | 1384 |  | 8.3 |
| *w*(CH_2_) – **B**-hexyl | 257 | 1385 |  | 40.6 | 256 | 1384 | 1396 | 66.8 |
| *ν_s_*(NO_2_), *ν*(С–NO_2_) – **D**/*ν*_s_(C_α_–C_β_), δ(∠C_α_NC_α_) – in-phase | 249 | 1358 | 1350 | 259.3 | 251 | 1360 | 1341 | 96.5 |
| *ν_s_*(NO_2_), *ν*(С–NO_2_) – **C, D** – out-of-phase/*ν*_s_(C_α_–C_β_), δ(∠C_α_NC_α_) – in-phase | 248 | 1357 |  | 980.9 | 250 | 1358 |  | 1030.1 |
| *ν*_s_(C_α_–C_β_), δ(∠C_α_NC_α_) – **I**, **III**_pyr. ring_, out-of-phase/*ν*_as_(C_α_–C_β_),*ν*_as_(C_α_ –N) – **II**, **IV**_pyr. ring_ | 247 | 1349 |  | 106.8 | 249 | 1358 |  | 89.5 |
| *ν*_s_(C_α_–C_β_) – **II**, **IV**_pyr. ring_, out-of-phase**/***ν*_as_(C_α_–C_β_), δ(C_β_H) – **I**, **III**_pyr. ring_ (*ZnTPP – ν*_s_(C_α_–C_β_) – **I**–**IV**_pyr. ring_**,** δ(∠C_α_C_m_C_α_) – in phase) | 246 | 1344 |  | 63.1 | 248 | 1353 |  | 0.2 |
| (*ZnTPP* – *ν*_s_(C_α_–C_β_) – **I**, **II** and **III**, **IV**_pyr. ring_ – out-of-phase/*ν*_as_(C=C)_benz_. – **C, D**_benz. ring_) | – | – | – | – | 245 | 1335 |  | 119.8 |
| (*ZnTPP* – *ν*_s_(C_α_–C_β_) – **I**, **III** and **II**, **IV**_pyr. ring_ – out-of-phase/*ν*_as_(C=C)_benz_ – **C, D**_benz. ring_) | – | – | – | – | 244 | 1335 |  | 52.7 |
| *ν*_as_(C_α_ –N)/δ(NH)/ δ(C_β_H)/*ν*_as_(C=C)_benz_ – **C,** **D** | 241 | 1319 | 1308 | 18.9 | 241 | 1318 |  | 41.1 |
| *ν*_as_(C=C) – **A**, **B**_benz. ring_ – in-phase/*ν*_s_(C_α_–C_β_), *ν*_s_(C_α_ –N) – **II**, **IV**_pyr. ring_ **/**δ(NH)/δ(C_β_H) | 240 | 1311 |  | 107.5 | 240 | 1307 | 1304 | 178.5 |
| *ν*_as_(C=C) – **A**, **B**_benz. ring_ – out-of-phase/*ν*_as_(C_α_–C_β_)/*ν*(C_α_ –N)/δ(C_β_H) | 239 | 1306 |  | 22.1 | 239 | 1305 |  | 0.5 |
| δ(NH)/τ(C_β_H)/*ν*_as_(C=C) – **A**_benz. ring_ (*ZnTPP* – *and* *ν*(C_α_–C_β_) – out-of phase/ *ν*(C*_m_*–C_benz_ **_A_**, **_B_**)) | 238 | 1302 |  | 19.8 | 238 | 1297 |  | 19.9 |
| τ(CH)_ph_ – **A**, **B**_benz. ring_**/***ν*_s_(C_α_–C_β_) – **I**, **III**_pyr. ring_ – in-phase/*ν*_s_(C_α_–N) – **I**, **III** and **II**, **IV**_pyr. ring_**,** out-of-phase (*ZnTPP* – *ν_s_*(C_α_–N) – **I, IV** and **II**, **III**_pyr. ring_ – out of phase) | 237 | 1298 | 1287 | 50.8 | 237 | 1292 |  | 0.9 |
| *w*(CH_2_) – **A**-hexyl | 232 | 1281 |  | 31.7 | 231 | 1280 | 1283 | 3.8 |
| *w*(CH_2_) – **B**-hexyl/τ(CH)_ph_ – **B**_benz. ring_ | 231 | 1279 |  | 35.2 | 230 | 1280 |  | 44.0 |
| *ν_as_*(C_benz_ **_B_**–O–C)/**A**_benz_-ring deformation | 223 | 1255 | 1246 | 306.3 | – | – | – | – |
| *ν_as_*(C_benz_ **_A_**–O–C)/**B**_benz_-ring deformation (*ZnTPP – and* **A**_benz_-ring deformation) | 222 | 1253 |  | 533.3 | 222 | 1253 | 1244 | 8.3 |
| *ν*(C_benz_–O) – **A**, **B** | 221 | 1251 |  | 386.7 | 221 | 1252 |  | 1234.8 |
| *w*(CH_2_) – **A**-hexyl | 216 | 1215 |  | 17.4 | 214 | 1214 | 1206 | 15.5 |
| *w*(CH_2_) – **B**-hexyl | 215 | 1214 | 1177 | 17.9 | 213 | 1214 |  | 18.5 |
| *ν*_as_(C_α_–C_β_), δ(C_β_H) – **I**, **III**_pyr. ring_ **/II**, **IV –** pyr. ring breathing | 214 | 1210 |  | 30.4 | 212 | 1198 |  | 23.7 |
| *ν*(C*_m_*–C_benz_)/δ(CH)/δ(C_β_H)/**I**, **III –** pyr. ring breathing, out-of-phase (*ZnTPP – and* **II*,* IV** *–* pyr. ring breathing, out-of-phase) | 213 | 1205 |  | 8.2 | 211 | 1195 |  | 42.2 |
| δ(NH) | 209 | 1177 |  | 16.3 | – | – | – | – |
| *ν*_as_(C_α_ –N) – **II**, **IV**_pyr. ring_ **/***ν*_s_(C_α_–C_β_), δ(C_β_H), δ(∠C_α_NC_α_) – **I**, **III**_pyr. ring_, out-of-phase (*ZnTPP – ν*_as_(C_α_ –C_β_) – **I–IV**_pyr. ring_ **/**δ(C_β_H)) | 208 | 1164 |  | 43.1 | 208 | 1162 |  | 0.01 |
| δ(CH)_ph_ | 205 | 1157 |  | 56.0 | 205 | 1156 | 1177 | 5.6 |
| δ(CH)_ph_ | 204 | 1156 |  | 186.5 | 204 | 1156 |  | 229.9 |
| δ(CH)_ph_ – **C/C**_benz_-ring deformation | 196 | 1095 | 1109 | 32.4 | 197 | 1095 | 1109 | 30.2 |
| δ(CH)_ph_ – **D/D**_benz_-ring deformation | 195 | 1095 |  | 66.9 | 196 | 1094 |  | 63.8 |
| δ(CH)_ph_ – **C**_benz. ring_ | 194 | 1084 | 1074 | 9.5 | 195 | 1084 | 1072 | 9.5 |
| δ(CH)_ph_ – **D**_benz. ring_ | 193 | 1083 |  | 11.1 | 194 | 1083 |  | 10.9 |
| *ν*(C–C) – **A**-hexyl | 187 | 1048 | 1057 | 32.0 | 187 | 1048 | 1047 | 4.9 |
| *ν*(C–C) – **B**-hexyl | 186 | 1047 |  | 35.8 | 186 | 1047 |  | 58.8 |
| *ν*(C_hex_–O)/*ν*(C–C) – **A**-hexyl | 184 | 1032 | 1017 | 62.6 | 185 | 1036 | 1030 | 2.1 |
| *ν*(C_hex_–O)/*ν*(C–C) – **B**-hexyl | 183 | 1032 |  | 89.7 | 184 | 1032 |  | 134.1 |
| *ν*(C_hex_–O)/*ν*(C–C) – **A**-hexyl | 182 | 1028 |  | 14.6 | 183 | 1029 |  | 1.3 |
| *ν*(C_hex_–O)/*ν*(C–C) – **B**-hexyl | 181 | 1027 |  | 13.7 | 182 | 1027 |  | 12.7 |
| (*ZnTPP –* **I, IV** and **II, III –** pyr. ring breathing, out-of-phase/**C**, **D**_benz. ring_ – ring deformation) | – | – | – | – | 173 | 994 | 997 | 133.6 |
| (*ZnTPP –* **I, II** and **III, IV –** pyr. ring breathing, out-of-phase/**A**, **B**_benz. ring_ – ring deformation) | – | – | – | – | 170 | 989 |  | 118.0 |
| **I**, **III –** pyr. ring breathing | 171 | 982 | 982 | 67.4 | – | – | – | – |
| γ(CH)_ph_ /**II**, **IV –** pyr. ring breathing, out-of-phase | 162 | 964 | 968 | 115.8 | – | – | – | – |
| γ(CH)_ph_ – **C**, **D**_benz. ring_/pyr. ring deformation | 147 | 876 | 866 | 10.8 | 153 | 884 | 866 | 9.1 |
| γ(CH)_ph_ /*ν*(C*_m_*–C_benz_ **_C_**), *ν*(C*_m_*–C_benz_ **_D_**) – out-of-phase/pyr. ring deformation | 146 | 872 |  | 39.9 | 147 | 877 |  | 17.6 |
| γ(CH)_ph_ – **A**, **B**_benz. ring_ | 141 | 845 | 849 | 125.3 | 142 | 847 | 851 | 119.6 |
| γ(CH)_ph_ | 139 | 838 |  | 49.0 | 140 | 838 |  | 80.5 |
| γ(CH)_ph_ – **A**, **B**_benz. ring_ | 136 | 814 |  | 0.9 | 136 | 814 | 820 | 29.0 |
| γ(CH)_ph_ – **A**, **B**_benz. ring_ | 135 | 813 |  | 11.0 | 135 | 814 |  | 10.9 |
| γ(CH)_ph_ /pyr. ring deformation | 132 | 810 |  | 0.5 | 131 | 805 | 797 | 14.4 |
| γ(CH)_ph_ /pyr. ring deformation | 131 | 807 | 800 | 14.7 | 129 | 800 |  | 52.9 |
| γ(C_β_H)/γ(CH)_ph_ – **B**_benz. ring_ | 130 | 804 |  | 83.0 | 128 | 796 |  | 0.9 |
| γ(NH)/γ(C_β_H)/γ(CH)_ph_ | 118 | 740 |  | 32.8 | 118 | 740 | 745 | 36.6 |
| γ(NH)/γ(C_β_H)/γ(CH)_ph_ | 117 | 739 |  | 4.1 | 116 | 724 | 719 | 19.3 |
| γ(NH) | 116 | 730 | 731 | 54.2 | – | – | – | – |
| γ(CH)_ph_ – **C, D**_benz. ring_ | 106 | 694 |  | 6.6 | 109 | 696 | 700 | 11.9 |
| macrocycle – out-of-plane | 105 | 677 |  | 3.2 | 107 | 679 |  | 2.2 |
| **A, B**_benz_ – ring deformation/macrocycle – out-of-plane | 100 | 633 | 631 | 14.6 | 103  101 | 635  628 | 633 | 17.0  29.4 |
| **A, B**_benz_ – ring deformation | 98 | 625 |  | 6.7 | 98 | 624 |  | 0.1 |
| δ(∠C_benz_ **_A_**–O–C)/δ(∠C_benz_ **_B_**–O–C)/**A**, **B**_benz_–ring deformation | 97 | 624 |  | 48.3 | 99 | 626 |  | 25.6 |

* *ν –* stretching vibration; deformation vibrations: δ(*b*∠) – scissoring, *w –* wagging*,* τ – twisting; γ – out-of-plane vibrations.

**Table S4.** Calculated and experimental wavenumbers (*ν,* cm^–1^) and IR absorption intensity (*I*, km/mole) of the normal vibrations of the 5,10, 15, 20-*tetrakis*(4-hexoxyphenyl)-porphine and of the complex 5,10, 15, 20-*tetrakis*(4-hexoxyphenyl)-porphine with Zinc in the 1650–600 cm^–1^ range.

| Compound | **1** (TPP) | | | | **4** (ZnTPP) | | | |
| --- | --- | --- | --- | --- | --- | --- | --- | --- |
| Assignments (*cursive in parenthesis corresponds to ZnTPP*) | Mode | *ν_calc._* | *ν_exp._* | *I*_abs_. | Mode | *ν_calc._* | *ν_exp._* | *I*_abs_. |
| *ν_s_*(C=C) – **A, B**_benz. ring_ – out-of-phase | 377 | 1619 | 1607 | 210.7 | 376 | 1619 | 1609 | 192.2 |
| *ν_s_*(C=C) – **C, D**_benz. ring_ **–** in phase | 376 | 1618 |  | 63.9 | 375 | 1618 |  | 72.8 |
| *ν_s_*(C=C) – **C, D**_benz. ring_ – out-of-phase | 375 | 1617 |  | 176.8 | 374 | 1618 |  | 163.5 |
| *ν_s_*(C=C) – **A – D**_benz. ring_ – out-of-phase | 373 | 1573 | 1572 | 22.8 | 372 | 1573 | 1574 | 33.7 |
| *ν_s_*(C=C) – **C, D**_benz. ring_ **–** in phase | 372 | 1573 |  | 20.8 | 371 | 1573 |  | 17.9 |
| δ(NH)/*ν*(C_β_–C_β_) – **II**, **IV**_pyr. ring_ – out-of-phase/*ν_as_*(C*_m_*–С_α_) | 370 | 1564 | 1560 | 79.6 | – | – | – | – |
| *ν*(C_β_–C_β_), *ν_s_*(С_α_–N) – **I**, **III** – out-of-phase | 365 | 1519 | 1518 | 38.4 | 366  367 | 1533  1533 | 1530 | 37.7  42.5 |
| *ν_as_*(C=C) – **A, B**_benz_ _ring_/*ν*(C*_m_*–C _benz_) – **A, B –** out-of-phase/*ν(*C_benz_–O) – **A, B –** out-of-phase | 363 | 1503 | 1508 | 300.3 | 365 | 1507 | 1512 | 197.5 |
| *ν_as_*(C=C) – **A, B**_benz_ _ring_/*ν*(C*_m_*–C _benz_) – **A, B –** in phase/*ν(*C_benz_–O) – **A, B –** in phase | 362 | 1503 |  | 42.0 | 362 | 1505 | 1497 | 5.5 |
| *ν_as_*(C=C) – **C, D**_benz ring_/*ν*(C*_m_*–C_benz_) – **C, D –** out-of-phase/*ν(*C_benz_–O) – **C, D –** out-of-phase | 361 | 1499 |  | 296.5 | 363 | 1505 |  | 165.0 |
| *ν_as_*(C=C) – **C, D**_benz. ring_/*ν*(C*_m_*–C_benz_) – **C, D –** in phase/*ν(*C_benz_–O) – **C, D –** in phase | 360 | 1499 |  | 26.3 | 360 | 1501 |  | 12.8 |
| δ(NH)/**II**, **IV**_pyr. ring_ **–** breathing/*ν_s_*(C*_m_*–С_α_)/*ν_as_*(С_α_–N) – **I**, **III**_pyr. ring_ | 359 | 1482 | 1468 | 80.0 | – | – | – | – |
| **I**, **III**_pyr. ring_ – breathing **–** out-of- phase/*ν_as_*(C_α_ –С*_m_*) (*ZnTPP – and*  **II**, **IV**_pyr. ring_ ***–*** breathing **–** out-of-phase) | 358 | 1478 |  | 235.6 | 359  358 | 1490  1488 | 1468 | 235.5  314.4 |
| δ(CH_2_) **– A, B**-hexyl – out-of-phase | 356 | 1459 |  | 99.7 | 355 | 1459 | 1462 | 75.4 |
| δ(CH_2_), δ(CH_3_) **– B**-hexyl | 343 | 1432 |  | 2.5 | 341 | 1432 | 1433 | 4.6 |
| δ(CH_2_), δ(CH_3_) **– A**-hexyl | 342 | 1432 |  | 2.9 | 340 | 1432 |  | 4.8 |
| δ(CH_2_), δ(CH_3_) **– C**-hexyl | 336 | 1431 | 1429 | 5.5 | 334 | 1431 |  | 5.7 |
| *ν_as_*(C=C) – **A, B**_benz. ring_**/**δ(CH)_ph_ | 327 | 1404 |  | 12.9 | 325 | 1404 |  | 8.2 |
| *w*(CH_2_) – **B**-hexyl | 322 | 1384 | 1377 | 35.3 | 321 | 1385 | 1379 | 34.2 |
| *w*(CH_2_) – **A**-hexyl | 321 | 1384 |  | 39.9 | 320 | 1384 |  | 44.5 |
| *w*(CH_2_) – **C, D**-hexyl | 319 | 1375 |  | 23.1 | 319 | 1375 |  | 13.6 |
| *w*(CH_2_) – **C, D**-hexyl/*ν*_as_(C_α_–N) – **I**, **III**_pyr. ring_/*ν*_as_(C_α_–C_β_) – **I**, **III/**δ(∠C_α_C*_m_*C_α_) | 318 | 1374 |  | 4.8 | 318 | 1375 |  | 23.4 |
| δ(NH)/*ν*_as_(C_α_–N) – **I**, **III**_pyr. ring_**/***ν*_s_(C_α_–N) – **II**, **IV/***ν*_as_(C_α_–C_β_) – **I**–**III/***ν*_s_(C_α_–C_β_) – **II**, **IV**_pyr. ring_ | 309 | 1362 |  | 50.2 | 309 | 1358 |  | 0.6 |
| *ν*_s_(C_α_–C_β_) – **I**, **III**_pyr. ring_ – out-of-phase/*ν_as_*(C_α_–N) – **II, IV**_pyr. ring_ | 307 | 1349 | 1352 | 111.6 | 303 | 1334 | 1341 | 121.8 |
| *ν*_s_(C_α_–C_β_) – **II, IV**_pyr. ring_ – out-of-phase/δ(NH)/*ν_as_*(C_α_–N) – **I**–**III /***ν_as_*(C_α_–C_β_) – **I**, **III**_pyr. ring_ | 306 | 1343 |  | 81.8 | 302 | 1333 |  | 89.9 |
| *w*(CH_2_), *w*(CH_3_) – **A, B**-hexyl | 304 | 1339 |  | 34.3 | 307 | 1340 |  | 15.6 |
| *ν_as_*(C=C) – **C, D**_benz. ring_**/**δ(C_β_H) | 300 | 1311 | 1302 | 153.2 | 300 | 1310 | 1304 | 172.9 |
| *ν_as_*(C=C) – **C, D**_benz. ring_ | 299 | 1309 |  | 35.9 | 299 | 1308 |  | 67.8 |
| *ν_as_*(C=C) – **A, B**_benz. ring_ | 297 | 1305 |  | 173.1 | 297 | 1305 |  | 149.5 |
| δ(C_β_H)/δ(NH) | 296 | 1303 |  | 32.9 | 296 | 1294 |  | 11.3 |
| *ν*_s_(C_α_–C_β_) – **II, IV**_pyr. ring_ –in phase *ν*_as_(C_α_–N) – **I**, **III** – in phase /*ν_s_*(C=C) – **A, B**_benz. ring_ | 295 | 1298 |  | 18.8 | 295 | 1294 |  | 13.5 |
| *w*(CH_2_) – **A, B**-hexyl/δ(CH) – **A, B** _benz. ring_ (*ZnTPP and* τ(CH_2_) *–* **C, D***-*hexyl) | 286 | 1282 | 1285 | 48.5 | 286 | 1284 |  | 1.2 |
| *w*(CH_2_) – **C**-hexyl | 285 | 1281 |  | 13.3 | 282 | 1281 | 1285 | 18.2 |
| *w*(CH_2_) – **D**-hexyl | 284 | 1281 |  | 13.1 | 285 | 1282 |  | 12.1 |
| *w*(CH_2_) – **A, B**-hexyl | 283 | 1281 |  | 7.2 | 284 | 1281 |  | 28.3 |
| *w*(CH_2_) – **A, B**-hexyl **/**δ(CH)_ph_ – **A, B**_benz. ring_ | 282 | 1280 |  | 2.5 | 283 | 1281 |  | 28.3 |
| δ(NH)/*ν*(C*_m_*–C_benz_)/*ν*(C_benz_–O) – **A, D**/τ(CH_2_) – **D**-hexyl | 273 | 1254 | 1246 | 226.9 | 272 | 1253 |  | 48.6 |
| *ν*(C*_m_*–C_benz_. **_B_**)/*ν*(C_benz_. **_B_**–O)/τ(CH_2_) – **B-**hexyl | 272 | 1253 |  | 105.9 | – | – | – | – |
| δ(NH)/*ν*(C*_m_*–C_benz_. **_A–D_**)**/***ν*(C_benz_. **_A–D_** –O) –out-of-phase/τ(CH_2_) – **C, D**-hexyl (*ZnTPP – ν*(C*_m_*–C_benz_. **_A–D_**) – disappear) | 271 | 1252 |  | 241.9 | 271 | 1252 | 1246 | 264.5 |
| *ν*(C_benz_. **_A, B_**–O)– out-of-phase/δ(CH) _ph_ – **A, B**_benz. ring_ | 269 | 1249 |  | 986.1 | 269 | 1250 |  | 1219.7 |
| *ν*_as_(C_α_–N) – **II**, **IV**_pyr. ring_ **/**δ(C_β_H) – **I**– **IV**_pyr. ring_ **/**δ(CH) **– A**–**D**_benz. ring_ (*ZnTPP –* δ(CH)_ph_ – **A**–**D** *and ν*_as_(C_α_–N) – **I–** **IV**_pyr. ring_ – disappear) | 268 | 1244 |  | 0.1 | 266 | 1236 |  | 36.7 |
| *ν_s_*(C=C) – **A, B**_benz. ring_**/***ν(*C_benz_–O) – **A, B** – in phase/τ(CH_2_) | 265 | 1234 |  | 25.0 | 265 | 1235 |  | 81.2 |
| *ν(*C_benz_–O) – **A, B** – out-of-phase/τ(CH_2_)/δ(CH)_ph_ – **A, B**_benz. ring_ | 264 | 1233 |  | 508 | 264 | 1234 |  | 417.9 |
| *w*(CH_2_), *w*(CH_3_)– **B**-hexyl | 262 | 1215 |  | 16.0 | 260 | 1215 |  | 15.8 |
| *w*(CH_2_), *w*(CH_3_) – **A**-hexyl | 261 | 1215 |  | 22.3 | 259 | 1215 |  | 21.7 |
| *w*(CH_2_), *w*(CH_3_) – **C**-hexyl | 260 | 1210 | 1211 | 45.4 | 257 | 1210 | 1207 | 52.6 |
| *w*(CH_2_), *w*(CH_3_) – **D**-hexyl | 259 | 1210 |  | 30.3 | 258 | 1210 |  | 42.3 |
| τ (C_β_H) – **I**, **III**_pyr. ring_**/***ν*(C*_m_*–C_benz_) – **I**, **III**_pyr. ring_ **–** out-of-phase | 258 | 1207 |  | 49.0 | 255 | 1196 |  | 32.8 |
| τ (C_β_H) – **II**, **IV**_pyr. ring_ /*ν*(C*_m_*–C_benz_) – **II**, **IV**_pyr. ring_ – out-of-phase | 257 | 1205 |  | 16.6 | 256 | 1196 |  | 35.0 |
| τ(CH_2_) – **C**-hexyl | 256 | 1192 |  | 11.0 | 254 | 1192 |  | 8.8 |
| τ(CH_2_) – **D**-hexyl | 255 | 1191 |  | 12.4 | 253 | 1192 |  | 15.6 |
| δ(NH) | 251 | 1173 |  | 12.2 | – | – | – | – |
| δ(CH) _ph_ **– A,B**_benz. ring_ | 246 | 1155 | 1175 | 220.7 | 246 | 1155 | 1177 | 204.1 |
| δ(CH) _ph_ **– A–D**_benz. ring_ /*r*(CH_2_) – **C-**hexyl | 245 | 1154 |  | 54.8 | 245 | 1154 |  | 66.3 |
| δ(CH) **– C,D**_benz. ring_**/***r*(CH_2_) – **D**-hexyl | 244 | 1153 | 1152 | 170.4 | 244 | 1153 | 1150 | 150.7 |
| δ(CCC) – **A**-hexyl | 240 | 1115 | 1107 | 26.2 | 240 | 1115 | 1109 | 25.7 |
| δ(CH)_ph_ **– B**_benz. ring_ | 236 | 1094 | 1070 | 11.0 | 237 | 1094 | 1072 | 8.9 |
| δ(CH)_ph_ **– A**_benz. ring_ | 235 | 1094 |  | 9.2 | 236 | 1094 |  | 8.5 |
| δ(CH)_ph_ **– C**_benz. ring_ | 234 | 1093 |  | 19.3 | 235 | 1093 |  | 24.5 |
| δ(CH)_ph_ **– D**_benz. ring_ | 233 | 1093 |  | 11.0 | 234 | 1093 |  | 7.9 |
| **C**-hexyl – rocking/δ(C_β_H) | 227 | 1050 |  | 9.9 | 228 | 1050 |  | 17.8 |
| δ(C_β_H) – **II**, **IV**_pyr. ring_– out-of-phase | 226 | 1049 | 1049 | 43.4 | 227 | 1049 | 1047 | 9.2 |
| δ(C_β_H) – **II**, **IV**_pyr. ring_– out-of-phase (*ZnTPP –* **I**, **III**_pyr. ring_– out-of-phase) | 224 | 1048 |  | 48.2 | 226 | 1049 |  | 43.0 |
| *ν*(C_hex_–O) – **A**-hexyl | 223 | 1042 | 1045 | 65.3 | – | – | – | – |
| *ν*(C_hex_–O) – **C**-hexyl (*ZnTPP –and* **D**-hexyl) | 222 | 1042 |  | 56.2 | 222  223 | 1042  1044 | 1042 | 58.3  48.1 |
| δ(C_β_H) – **I, III**_pyr. ring_– out-of-phase | 221 | 1039 |  | 4.0 | 224 | 1048 |  | 44.9 |
| *ν*(C_hex_–O) – **B**-hexyl (*ZnTPP –and* **A**-hexyl) | 220 | 1034 | 1032 | 19.7 | 221 | 1037 | 1034 | 6.6 |
| *ν*(C_hex_–O) – **A**-hexyl (*ZnTPP –and* **B**-hexyl) | 219 | 1034 |  | 116.0 | 220 | 1034 |  | 121.8 |
| *ν*(C_hex_–O) – **D**-hexyl | 218 | 1030 |  | 20.8 | 219 | 1032 |  | 4.7 |
| *ν*(C_hex_–O) – **C**-hexyl (*ZnTPP –and* **D**-hexyl) | 217 | 1030 |  | 21.3 | 218 | 1031 |  | 18.1 |
| *ν_as_*(C–C) – **D-**hexyl (*ZnTPP –and* **C**-hexyl) | 214 | 1013 | 1017 | 16.3 | 212 | 1013 |  | 3.6 |
| *ν_as_*(C–C) – **C-**hexyl (*ZnTPP –* **C, D**-hexyl/δ(C_β_H)) | 213 | 1013 |  | 18.2 | 211 | 1011 | 1018 | 35.3 |
| *ν*(C–C) – **A, B-**hexyl | 206 | 989 | 991 | 12.5 | 200 | 989 |  | 24.8 |
| δ(C_β_H) – **II**, **IV**_pyr. ring_– out-of-phase | 204 | 987 |  | 10.4 | – | – | – | – |
| **I, III**_pyr. ring_ **–** breathing **–** out-of-phase (*ZnTPP –* *and* **II**, **IV**_pyr. ring_ **–** out-of-phase) | 201 | 980 | 982 | 102.3 | 202  203 | 989  990 | 1001 | 97.6  122.0 |
| **II**, **IV**_pyr. ring_ **–** breathing – out-of-phase | 195 | 964 | 968 | 121.1 |  |  |  |  |
| γ(CH)_ph_ – **D**_benz. ring_ (*ZnTPP –* **A**–**D**_benz. ring_) | 189 | 955 | 936 | 9.5 | 190 | 954 | 939 | 0.9 |
| γ(CH)_ph_ – **A**–**D**_benz. ring_ (*ZnTPP –* **A, B**_benz. ring_) | 167 | 843 | 841 | 18.6 | 168 | 845 | 849 | 12.5 |
| γ(CH)_ph_ – **A**–**D**_benz. ring_ (*ZnTPP –* **C, D**_benz. ring_) | 166 | 841 |  | 0.1 | 167 | 844 |  | 10.5 |
| γ(CH)_ph_ – **A**–**D**_benz. ring_ | 165 | 841 |  | 24.6 | 166 | 841 |  | 0.2 |
| γ(CH)_ph_ – **A**–**D**_benz. ring_ | 163 | 812 |  | 11.7 | 164 | 813 | 818 | 14.2 |
| γ(CH)_ph_ – **A**–**D**_benz. ring_ (*ZnTPP –* **A, B**_benz. ring_) | 159 | 810 |  | 2.9 | 158 | 805 | 804 | 42.2 |
| γ(C_β_H)/γ(NH) | 158 | 804 | 804 | 102.3 | 157 | 802 |  | 53.9 |
| γ(C_β_H)/γ(CH)_ph_ – **A–D**_benz. ring_ (*ZnTPP –* γ(CH)_ph_ – only **A, B**_benz. ring_) | 156 | 800 |  | 17.0 | 155 | 799 |  | 39.5 |
| γ(CH)_ph_ – **A**–**D**_benz. ring_ /γ(C_β_H) – **I**–**IV**_pyr. ring_ | 148 | 787 | 791 | 32.0 | – | – | – | – |
| γ(NH) | 138 | 734 | 741 | 48.2 | – | – | – | – |
| macrocycle and **A, B**_benz. ring_ – out-of-plane (*ZnTPP – and* **C, D**_benz. ring_ – out-of-plane) | 136 | 726 |  | 0.03 | 137 | 727 | 723 | 12.7 |
| **A**– **D**_benz. ring_ (*ZnTPP –* **C**, **D**_benz. ring_) *–* deformation | 122 | 635 | 637 | 10.0 | 122 | 635 | 640 | 16.3 |
| **A**, **B**_benz. ring_ *–* deformation/δ(C**_A_** _benz. ring_OC), δ(C**_B_** _benz. ring_ OC) – out-of-phase | 119 | 625 | 629 | 40.7 | 120 | 628 | 635 | 46.8 |

* *ν –* stretching vibration; deformation vibrations: δ(*b*Ð) – scissoring, *w –* wagging*,* τ – twisting; γ – out-of-plane vibrations.
